# Exploring the Synergistic Effects of Erinacines on Microglial Regulation and Alzheimer's Pathology Under Metabolic Stress

**DOI:** 10.1111/cns.70137

**Published:** 2024-12-17

**Authors:** Van Thanh Bui, Kuan‐Wei Wu, Chin‐Chu Chen, Anh Thuc Nguyen, Wei‐Jan Huang, Li‐Ya Lee, Wan‐Ping Chen, Chi‐Ying Huang, Young‐Ji Shiao

**Affiliations:** ^1^ Institute of Biopharmaceutical Science, National Yang Ming Chiao Tung University Taipei Taiwan; ^2^ Taiwan National Graduate Program in Molecular Medicine Academia Sinica, National Yang Ming Chiao Tung University Taipei Taiwan; ^3^ Grape King Bio Ltd Taoyuan City Taiwan; ^4^ PhD. Program in Biotechnology Research and Development Taipei Medical University Taipei Taiwan; ^5^ Department of Biochemistry, College of Medicine Kaohsiung Medical University Kaohsiung Taiwan; ^6^ Chong Hin Loon Memorial Cancer and Biotherapy Research Center National Yang Ming Chiao Tung University Taipei Taiwan; ^7^ National Research Institute of Chinese Medicine Ministry of Health and Welfare Taipei Taiwan

**Keywords:** Alzheimer's disease, erinacines, *Hericium erinaceus*, metabolic stress, microglia, neuroinflammation

## Abstract

**Background:**

*Hericium erinaceus* mycelium and its constituents, erinacines A and S, have shown neuroprotective effects in APP/PS1 transgenic mice; however, the precise mechanisms by which they modulate microglial phenotypes remain unclear. Our study is the first to explore the effect of erinacines on microglia morphology and the underlying mechanisms using a novel primary mixed glia cell model and advanced bioinformatic tools. Furthermore, we emphasize the clinical relevance by evaluating erinacines in a metabolically stressed APP/PS1 mouse model, which more accurately reflects the complexities of human Alzheimer's disease (AD), where metabolic syndrome is a common comorbidity.

**Methods:**

Rat primary mixed glial cultures were used to simulate the spectrum of microglial phenotypes, particularly the transition from immature to mature states. Microarray sequencing, along with Connectivity Map, ConsensusPathDB, and Gene Set Enrichment Analysis, identified pathways influenced by erinacines. The therapeutic efficacy was further evaluated in metabolically stressed APP/PS1 mice.

**Results:**

Erinacines significantly promoted the development of a ramified, neuroprotective microglial phenotype. Bioinformatics revealed potential modulation of microglia via histone deacetylase inhibition, actin filament dynamics, and synaptic structure modification—pathways not previously linked to erinacines in AD. Importantly, erinacines significantly lower fasting blood glucose and insulin levels while reducing amyloid‐beta plaque burden, suppressing hyperactivated glial responses, and enhancing neurogenesis in the metabolically stressed APP/PS1 mice.

**Conclusion:**

Our findings demonstrate the dual action of erinacines in modulating microglia morphology and phenotype while providing neuroprotection in a model that closely mimic the complexities of human Alzheimer's disease. Additionally, this study provides the foundation for understanding the potential mechanisms of action of erinacines, highlighting their promise as a novel treatment approach for Alzheimer's, particularly in cases complicated by metabolic dysfunction.

## Graphical abstract

Erinacines enhance microglial ramification and alleviate Alzheimer's disease pathology in a metabolically stressed model. This study reveals novel mechanisms, including histone deacetylase inhibition, supporting erinacines as a promising therapeutic for Alzheimer's, particularly in cases complicated by metabolic dysfunction.

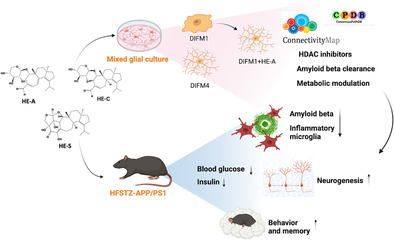

**Abbreviations**
AD: Alzheimer's disease
APP: amyloid precursor protein
APP/PS1: APPswe/PS1ΔE9
Aβ: amyloid β protein
BBB: blood–brain barrier
BrdU: 5‐bromo‐2′‐deoxyuriding
BSA: bovine serum albumin
CMap: connectivity map
CNS: central nervous system
DCX: doublecortin
DIFM: days in fresh medium
DIV: day in vitro
DMEM: Dulbecco's Modified Eagle's Medium
DMF: dimethyl fumarate
DMSO: dimethyl sulfoxide
ELISA: enzyme‐linked immunosorbent assay
FBS: fetal bovine serum
GFAP: glial fibrillary acidic protein
GSEA: Gene set enrichment analysis
HDAC: Histone deacetylase
HE‐A: erinacine A
HE‐C: erinacine C
HE‐Et: HE‐My ethanol extract
HE‐My: *Hericium erinaceus* mycelia
HE‐S: erinacine S
HFD: high fat diet
HFSTZ: HFD plus STZ
HOMO‐IR: Homeostasis Model Assessment‐Insulin Resistance
Iba‐1: ionized calcium‐ binding adaptor molecule 1
IFN‐γ: interferon γ
IL: interleukin
KEGG: Kyoto Encyclopedia of Genes and Genomes
LINCS: Integrated Network‐based Cellular Signatures
LPS: lipopolysaccharide
M‐CSF: macrophage colony‐stimulating factor
MTT: 3‐(4,5‐Dimethyl‐2‐thiazolyl)‐2,5‐diphenyl‐2H‐tetrazolium bromide
NB/B27: Neurobasal medium/B27 supplement
NCD: normal chow diet
NES: normalized enrichment score
OGTT: oral glucose tolerance test
PBS: phosphate‐ buffered saline
PC: perturbagen compound
PCL: perturbagen classes
PCR: polymerase chain reaction
PS1: presenilin 1
ROS: reactive oxygen species
SDS: sodium dodecyl sulphate
SGZ: subgranular zone
STZ: streptozotocin
TNF‐α: tumor necrosis factor‐α
WT: wild‐type

## 1 Introduction

Alzheimer's disease (AD) is a neurodegenerative disorder characterized by the accumulation of abnormal protein aggregates, such as beta‐amyloid (Aβ) plaques and tau tangles, leading to cognitive decline and memory loss. In addition to the classical proteinopathies, neuroinflammation and metabolic stress have emerged as critical and intertwined contributors to AD pathogenesis [1, 2, 3]. Neuroinflammation, characterized by the persistent activation of microglia and astrocytes, leads to the release of proinflammatory cytokines and reactive oxygen species (ROS). This inflammatory milieu creates a vicious cycle, recruiting more immune cells and exacerbating tissue damage, yet fails to effectively clear Aβ. Current treatments for AD primarily offer symptomatic relief, with cholinesterase inhibitors and NMDA receptor antagonists showing limited success in slowing disease progression.

In AD patients, metabolic comorbidities such as type 2 diabetes (T2DM) and hyperlipidemia (HLD) are significantly more prevalent than in the non‐AD population. It is estimated that up to 81% of AD patients also have type 2 diabetes [4]. Conversely, T2DM and HLD are known to increase the risk of AD by up to 50% [5, 6]. These findings highlight the importance of addressing metabolic dysfunction in AD patients, as this subpopulation may benefit from therapeutic approaches targeting both metabolic and neurodegenerative pathways. Although emerging therapies, such as monoclonal antibodies, target specific pathological features like amyloid‐beta, they do not address the underlying metabolic dysfunctions that are especially relevant in AD patients with comorbid metabolic stress. Moreover, conventional anti‐amyloid therapies have proven insufficient in resolving the neuroinflammatory cascade, underscoring the need for developing therapeutic strategies that directly target glial cell activity [7].

Microglia, the central nervous system (CNS)'s intrinsic immune cells, play a pivotal role in the homeostatic maintenance of brain functions and are intimately involved in AD pathogenesis [8]. Microglia migrate to the CNS during early embryonic development and remain there with the ability to self‐renew throughout life. During development, microglia are highly phagocytic and display an amoeboid morphology, which is essential for clearing apoptotic cells and debris [9]. In contrast, microglia in the adult CNS display a ramified morphology, indicative of a surveillance state where they exert neuroprotective functions [10, 11]. Maintaining or inducing this ramified state is crucial, as it enables microglia to efficiently monitor and respond to subtle changes in the CNS environment, thereby preventing unnecessary inflammation and providing support to neuronal health. On the other hand, upon encountering pathogens or injury, microglia undergo a phenotypic transformation back to an amoeboid form, enhancing their phagocytic activity [12, 13]. Although this activation is initially protective, chronic activation leads to microglial priming, rendering them hyper‐responsive to subsequent stimuli. This state of heightened reactivity fosters a spectrum of microglial phenotypes, driven by signaling molecules such as macrophage colony‐stimulating factor (M‐CSF) and interferon‐γ (IFN‐γ) [14, 15]. Sustained activation of microglia may potentiate neuronal injury [16, 17, 18]. Thus, reprogramming microglia from a detrimental to a protective state represents a promising therapeutic approach.

Metabolic dysfunction, particularly hyperglycemia, obesity, and insulin resistance, which are hallmarks of metabolic syndrome, also plays a crucial role in accelerating AD pathology [19, 20]. Transgenic mouse models have demonstrated that metabolic stress, induced by dietary manipulations such as a high‐fat diet (HFD) or streptozotocin (STZ) administration, exacerbates the Aβ plaque burden and cerebrovascular inflammation [21]. Our previous work established a metabolically stressed APP/PS1 transgenic mouse model (HFSTZ‐APP/PS1), where we found that a HFD combined with low‐dose STZ injections exacerbated Aβ pathology and astrocyte activation [22]. While normal APP/PS1 mice do not fully capture the complexity of AD, as occurs in humans with metabolic stress, the enhanced pathological features make metabolically stressed AD mice a more challenging and clinically relevant model for testing the efficacy of potential AD treatments. Therefore, studying the effects of drugs in metabolically stressed AD mice provides insights that are more applicable to real‐world clinical scenarios.

*Hericium erinaceus*, an edible and medicinal mushroom, has garnered attention for its neuroprotective properties, particularly in age‐related neurological disorders such as AD [23]. The mycelium of *Hericium erinaceus* (HE‐My) contains bioactive compounds known as erinacines, which include cyathane diterpenoids such as erinacine A (HE‐A), erinacine C (HE‐C), and the recently identified sesterterpene erinacine S (HE‐S) [24, 25]. These compounds, isomers with similar molecular weights (Figure S1), have been previously reported to have neuroprotective effects primarily through promoting nerve growth factor (NGF) synthesis and exhibiting anti‐inflammatory and antioxidant properties [26]. Our recent studies on APPswe/PS1ΔE9 (APP/PS1) transgenic mice have demonstrated that HE‐My and its constituents, particularly HE‐A and HE‐S, can modulate glial activity, leading to favorable alterations in AD pathology [27, 28]. However, the potential of erinacines to modulate microglia morphology and address metabolic dysfunction in the context of AD has not been explored.

In this study, we sought to elucidate the effects and the mechanisms of erinacines on microglial phenotype changes, both in vitro using a primary mixed glial culture and in vivo using the HFSTZ‐APP/PS1 mouse model. By examining microglial morphology, functional phenotypes, and gene expression in mixed glial cultures, alongside assessments of glycemic control, nesting behavior, Aβ burden, plaque‐associated microglial activation, and neurogenesis in HFSTZ‐APP/PS1 mice, we aimed to uncover novel mechanisms by which erinacines exert their neuroprotective effects. This investigation not only expands our understanding of erinacines in AD but also introduces a new dimension to their potential as therapeutic agents in neurodegenerative diseases complicated by metabolic stress.

## 2 Materials and Methods

### 2.1 Sample Preparation

Erinacines were prepared as described previously [27]. Briefly, at the end of the fermentation process, the mycelia were harvested, lyophilized, ground into powder, and stored in a desiccator at room temperature (i.e., HE‐My). HE‐My was extracted four times with 90% ethanol under reflux to obtain the ethanol extract (HE‐Et). Their chemical fingerprints were determined. Three active components, erinacine A (HE‐A), erinacine C (HE‐C), and erinacine S (HE‐S), in HE‐Et were identified. The structures of the erinacines are shown in Figure S1.

### 2.2 Cell Culture

Primary cultures of neonatal cortical glia were prepared from the cerebral cortex of Sprague–Dawley rat pups at postnatal Day 5 as described previously [29, 30]. The methodology adheres to protocols previously described, and practices were conducted in compliance with the ethical guidelines approved by the Institutional Animal Care and Use Committee at the National Research Institute of Chinese Medicine (IACUC number: 110‐417‐2). Briefly, neonatal pups were subjected to anesthesia through ether inhalation, carefully placed in a chilled dish, and subsequently euthanized via decapitation. The cerebral cortex was extracted to prepare a mixed glial cell culture, which was sustained in DMEM/F12 medium supplemented with 10% fetal bovine serum, 100 units/mL penicillin, and 100 μg/mL streptomycin. This culture was maintained for 3 days, after which the medium was replaced with a fresh DMEM/F12, and the cells were incubated for an additional 2 days. After that, the cells were cultured in Neurobasal medium supplemented with B27 (NB/B27), with the medium being refreshed every 3 days for long‐term culture.

To assess the differentiation and maturation processes of microglia within the glial cell mixture, cells were subjected to various durations of culture in fresh medium, resulting in distinct stages of microglial ramification. Specifically, NB/B27 was renewed at day in vitro (DIV)14 for the day in fresh medium (DIFM)4 microglial population and at DIV17 for the DIFM1 microglial subset, which, respectively, resulted in cultures conditioned in fresh medium for durations of 4 and 1 day by DIV18. To induce activation of the glial cells, lipopolysaccharide (LPS) (100 ng/mL) was added to the cultures at DIV17 and maintained for 24 h. The resulting morphological shifts in microglia and astrocytes were observed at DIV18.

### 2.3 Immunocytochemistry Assay

The cells were seeded in 24 well plates. At DIV17, the cells were treated with erinacines at the indicated concentrations for 24 h. For fluorescence observation, the cells were fixed with 4% paraformaldehyde in phosphate‐buffered saline (PBS) for 15 min at room temperature and the cells were washed twice with PBS. The cells were then permeabilized with 0.5% Triton X‐100 in PBS for 10 min and were blocked with 10% normal donkey serum (in PBS containing 0.5% bovine serum albumin, BSA) for 2 h at room temperature. Astrocytes and microglia were detected using glial fibrillary acidic protein (GFAP) antibody (Millipore Cat# AB5804, RRID:AB_2109645) and ionized calcium‐binding adaptor molecule‐1 (Iba‐1) antibody (Abcam Cat# ab5076, RRID:AB_2224402), respectively. Microglia phenotypes were detected using an anti‐CD86 antibody (BD Biosciences Cat# 553689, RRID:AB_394991), an anti‐CD206 antibody, (R and D Systems Cat# AF2535, RRID:AB_2063012), and an anti‐arginase 1 antibody (Cell Signaling Technology Cat# 9819, RRID:AB_2797712). Donkey anti‐goat IgG conjugated with DyLight649 and donkey anti‐mouse IgG conjugated with fluorescein were used as secondary antibodies.

### 2.4 Morphological Quantification of Microglia

MetaMorph software was used to calculate the cell area and convex area of microglia. The form factor was calculated by dividing the cell area with the convex area which has been described before [30, 31].

### 2.5 Measurement of Nitrite

To assess the amount of nitric oxide produced, the Griess reagent (0.05% N‐(1‐naphthyl)‐ethylene‐diamine dihydrochloride, 0.5% sulfanilamide, and 1.25% phosphoric acid) was employed. The accumulated nitrite, a stable breakdown product of nitric oxide, can be recorded. The optical density was detected at a wavelength of 540 nm using a microplate reader with NaNO_2_ as standard.

### 2.6 Cytokine Measurement

The levels of cytokines released in the cell culture supernatant, including tumor necrosis factor‐α (TNF‐α) and interleukin (IL)‐1β, were measured by specific enzyme‐linked immunosorbent assay (ELISA) kits following the manufacturer's instructions (R&D System, Minneapolis, MN).

### 2.7 Cell Viability Assay

A 3‐(4,5‐dimethyl‐2‐thiazolyl)‐2,5‐diphenyl‐2H‐tetrazolium bromide (MTT) assay was performed to evaluate the cell viability. Briefly, primary mixed glial cells (15.1 × 10^4^ cells/well) were inoculated in 24 well plates. At DIV17, the cells were treated with different concentrations of erinacines for 24 h. After 24 h, the cells were incubated with a medium containing 0.5 mg/mL MTT (Sigma M2128) for 30 min. The medium containing MTT was removed followed by the addition of dimethyl sulfoxide (DMSO) to dissolve the formazan particles. Colorimetric detection was measured at a wavelength of 600 nm by using an enzyme‐linked immunosorbent assay reader.

### 2.8 Connectivity Map (CLUE)

Five cell groups were used for the gene expression experiment: DIFM1 (group D1), DIFM4 (group D4), HE‐A‐treated DIFM1 (group A1), HE‐C‐treated DIFM1 (group C1), and HE‐S‐treated DIFM1 (group S1). The connectivity map (CMap) is a database that stores the gene expression profiles of numerous U.S. Food and Drug Administration‐approved drugs and other small molecules. The Library of Integrated Network‐based Cellular Signatures (LINCS) Unified Environment (CLUE), an extensive database developed from CMap, is a high‐throughput gene expression profiling technology. L1000 measures approximately 1000 validated landmark gene profiles in response to over 20,000 perturbations across many cultured human cells [32, 33].

In this study, groups D4; D1; A1, C1, and S1 were subjected to L1000 gene expression profiling. The process involved capturing mRNA transcripts from whole‐cell lysates via oligo‐dT. The resulting cDNAs were then amplified through ligation‐mediated polymerase chain reaction (PCR). The PCR amplicon was subsequently hybridized to fluorescent Luminex beads to assess the expression of specific genes. A *t*‐test rank order was used to identify significantly up‐ and downregulated probe sets. The predicted functional connections and the resulting gene expression pattern were compared with the CMap 2.0, available at Connectivity Map (CMAP) | Broad Institute.

### 2.9 Gene Set Enrichment Analysis (GSEA)

Gene set enrichment analysis (GSEA) was used to determine whether a predefined set of genes significantly differed among phenotypes [34] using GSEA 4.3.2 software [35]. The GSEA calculates the signal‐to‐noise ratio for the gene sets by normalized enrichment scores (NESs). We performed the GSEA with the default settings of the software, which included 1000 permutations, a phenotype permutation type, the exclusion of gene sets larger than 500 and smaller than 15 and the use of weighted enrichment statistics. Gene set databases (taken from MSigDB version 2022.1. Hs) used in this analysis included the Kyoto Encyclopedia of Genes and Genomes (KEGG), BioCarta, Pathway Interaction Database (PID), Reactome, and Wikipathways. The reference genes subjected to GSEA were erinacines vs. vehicle. Gene sets with a *p*‐value < 0.05 were considered significantly enriched.

### 2.10 Ethical Approval and Animal Management

The male APPswe/PS1ΔE9 double transgenic mouse model (APP/PS1) was purchased from Jackson Laboratory (No. 005864) that expressed a chimeric mouse/human APP695 harboring the Swedish K670M/N67L mutation and mutant human presenilin 1 (PS1) with the exon‐9 deletion mutation both under the control of the mouse prion protein promoter resulting in abundant amyloid plaques in cortex and hippocampus [36]. The animals were housed at a controlled room temperature (24 ± 1°C) and humidity (55%–65%) with a 12:12‐h (07:00–19:00) light–dark cycle. Experiments were conducted using male APP/PS1 transgenic mice and their wild‐type (WT) littermates. The Institutional Animal Care and Use Committee at the National Research Institute of Chinese Medicine approved the animal protocol (109‐417‐1 and 110‐417‐1).

### 2.11 Metabolic Stress Induction and Administration

HFSTZ‐induced mice were generated via HFD feeding and single‐dose STZ injecting [37]. Briefly, starting at the age of 10 weeks, the mice were fed either a normal chow diet (NCD; MF‐18, Oriental Yeast Co Ltd., Tokyo, Japan, *n* = 6) or an HFD (60% energy from fat; TestDiet, St. Louis, MO, USA) with water *ad libitum*. Mice on an HFD also received intraperitoneal injections of streptozotocin (50 mg/kg) after the mice had been fed with HFD for 2 weeks (HFSTZ group). The HFD was continued until the mice were killed after 8 weeks of dietary manipulation. To evaluate the ability of erinacines to ameliorate the impairment in HFSTZ mice, vehicle or erinacines (30 mg/kg/day) were orally administrated for 8 weeks after 10 weeks of HFD consumption (*n* = 6 for each group). The body weight was recorded every week after the dietary manipulations.

For neurogenesis measurement, 5‐bromo‐2′‐deoxyuridine (BrdU) was injected intraperitoneally at 50 mg/kg/day during the last 7 days.

### 2.12 Nesting Behavior

After individually housing mice for 5 h, a Nestlet pressed‐cotton square (Ancare, Canterbury, Kent, UK) was placed into each cage 1 h before the start of the dark cycle. After 16 h, nest construction was scored using a 5‐point scaling system [38]. A score of 1 signifies a nestlet that has been disturbed but nesting material has not been gathered to a nest site in the cage; a score of 2 indicates a flat nest; a score of 3 corresponds to a nest with a cup shape; a score of 4 indicates a dome that is not fully formed; and a score of 5 denotes a fully formed dome with an enclosed structure.

### 2.13 Blood Glucose Analysis and Oral Glucose Tolerance Test

Oral glucose tolerance tests (OGTTs) were performed after fasting for 16 h. The mice were given a solution containing glucose (3 g/kg) by oral gavage. Blood is drawn at intervals of 30 min for measurement of glucose levels. Blood glucose was measured using a glucometer (Bioptik Technology, Taipei, Taiwan).

### 2.14 Tissue Sample Collection

The mice were deeply anesthetized via intraperitoneal injection of 80 mg/kg sodium pentobarbital. Blood samples were collected by the cardiac puncture and then sacrificed by transcardial perfusion with 50 mL of saline. The brain was collected. One hemisphere was subjected to mechanical disruption in a homogenization buffer composed of 20 mM Tris–HCl at pH 7.4, 320 mM sucrose, 2 mM EDTA, 1 mM PMSF, 5 μg/mL leupeptin, and 5 μg/mL aprotinin. The remaining hemisphere was fixed in 4% formaldehyde at 4°C overnight and subsequently underwent cryoprotection. Thereafter, the brain tissue was cut into 30 μm thick sections. For staining and analytical assessment, three slides, collectively spanning the bregma coordinates from −1.58 to −1.82, were selected from each brain.

### 2.15 Serum Analysis

Serum insulin levels were determined with an insulin‐Kit HTRF (Cisbio, Codolet, France). The fluorescence intensities were measured on a SpectraMax M5 microplate reader (Molecular Devices, Sunnyvale, CA, USA). Homeostasis Model Assessment‐Insulin Resistance (HOMA‐IR) (fasting blood glucose [mM] × fasting insulin [U/mL]/22.5) scores were then calculated.

The concentration of Aβ was quantified using previously established methods [28]. In summary, cortical homogenates were combined in equal amounts with a homogenization buffer that included 4% SDS and protease inhibitors. This mixture was then subjected to sonication and subsequently centrifuged at 100,000 × *g* for 1 hour at a temperature of 4°C. The pellet (defined as SDS‐insoluble fraction) was re‐suspended in 70% formic acid and then centrifuged at 100,000 × *g* for 1 h at 4°C. The supernatant (defined as SDS‐soluble fraction) was collected and neutralized with Tris buffer (1 M, pH 11). Aβ levels were measured by human Aβ1–40 and Aβ1–42 Enzyme‐Linked Immunosorbent Assay (ELISA) kits (Invitrogen, KHB3482 and KHB3442). The detailed experiments were performed according to the manufacturer's protocol.

### 2.16 Amyloid Plaque Staining

Amyloid plaques were stained using Thioflavin S (ThS) and Amylo Glo. For ThS staining, ThS powder was dissolved in water at a ratio of 1%, which was then filtered through a 0.22 μm filter. Tissue sections were incubated in this solution for 60 min, protected from light, followed by two washed with 70% alcohol for 5 minutes each. The sections were then incubated in PBS solution for 5 min. For Amylo Glo staining, the 100X Amylo‐Glo stock solution was diluted with 0.9% NaCl. After completing the staining, the following tissue immunofluorescence staining was performed.

### 2.17 Immunohistochemistry Assay

Immunohistochemistry was performed as described previously [28]. Briefly, sections were blocked in blocking buffer (PBS containing 1% BSA, and 0.3% Triton X‐100) containing 3% normal donkey serum for 1 h. Then, the sections were incubated in blocking buffer containing 1% normal donkey serum and primary antibodies, including mouse monoclonal antibodies to Aβ1–16 (AB10, Millipore Cat# MAB5208‐100UG, RRID:AB_827114), and (Millipore Cat# AB5804, RRID:AB_2109645) and ionized calcium‐binding adaptor molecule‐1 (Iba‐1) antibody (Abcam Cat# ab5076, RRID:AB_2224402) overnight at 4°C. The sections were then incubated in blocking buffer containing 1% normal donkey serum and Hoechst 33258 (Invitrogen, Carlsbad, CA, USA, 2 μg/mL), fluorescein isothiocyanate‐, rhodamine red X‐ or Alexa Fluor 647‐conjugated donkey anti‐mouse IgG, anti‐rabbit IgG or anti‐goat IgG (Jackson ImmunoResearch, West Grove, PA, USA) at room temperature for 2 h. After being washed with PBS containing 0.01% Triton X‐100, the sections were mounted with Aqua Poly/Mount (Polyscience Inc., Warrington, PA, USA) for microscopic analysis using a Zeiss LSM 780 confocal microscopy (Jena, Germany). Representative confocal images are shown at a 10‐μm depth with maximal projection. The quantification of amyloid plaques was performed via ImageJ software.

### 2.18 Quantification of Immunofluorescence Intensity

Fluorescence images of GFAP and Iba‐1 double immunostaining with Amylo‐Glo‐stained senile plaques were taken at 40x magnification. The relative fluorescence intensity of GFAP and Iba‐1 in the vicinity of plaques was quantified using MetaMorph software (reference region: none plaque‐associated area; threshold level: 30–255 without shade correction). Confocal images of representative Amylo‐Glo‐stained senile plaques were of a 10‐mm depth with maximal projection. Binary images were converted by fluorescence intensity (threshold level: 20–255). Feret's diameter and plaque size were calculated based on binary images. The diameter of circled areas surrounding irregular‐shaped plaques was delimited by 8 times the Feret's diameter. The areas of overlapping plaques were excluded from the analysis. Two serial sections including the middle region of the hippocampus (−1.58 to −1.94 mm relative to the bregma) were analyzed for each mouse.

### 2.19 Neurogenesis

Neurogenesis was detected by incubating tissue sections in sodium citrate buffer (10 mM, pH 6.0, 80°C) for 30 min and 2 M HCl at 37°C for 30 min, and then in blocking buffer and antibody buffer with primary antibodies and corresponding secondary antibodies, as mentioned above. Primary antibodies include mouse anti‐BrdU antibody (Santa Cruz, CA, USA, sc‐32,323) and rabbit anti‐doublecortin (DCX) antibody (Abcam, ab18723, GR140153‐1). The number of BrdU‐ or DCX‐positive cells in the subgranular zone (SGZ) was quantified, and the linear density of BrdU‐ or DCX‐positive cells per millimeter of SGZ was calculated. The dendritic complexity of newborn neurons in the dentate gyrus was analyzed by laminar quantification of disjointed dendrites. The number of DCX‐positive cell bodies (a), primary (b) and secondary (c) dendrites were counted. The levels of primary dendrite sprouting and secondary dendritic branches were represented in b/a and c/b respectively.

### 2.20 Statistical Analysis

Statistical analysis was performed using GraphPad Prism (GraphPad, San Diego, CA, USA) and SPSS (IBM, New York, NY, USA). All values are reported as the mean standard error of the mean, derived from three or more independent assays. Normality of the data was assessed using the Shapiro–Wilk test in GraphPad Prism. In general, a one‐way analysis of variance was followed by post hoc analyses using Tukey's honest significant difference tests. A *p*‐value < 0.05 was considered statistically significant.

## 3 Results

### 3.1 Microglial Ramification and Maturation Vary on Different Days in Fresh Medium

**FIGURE 1:**
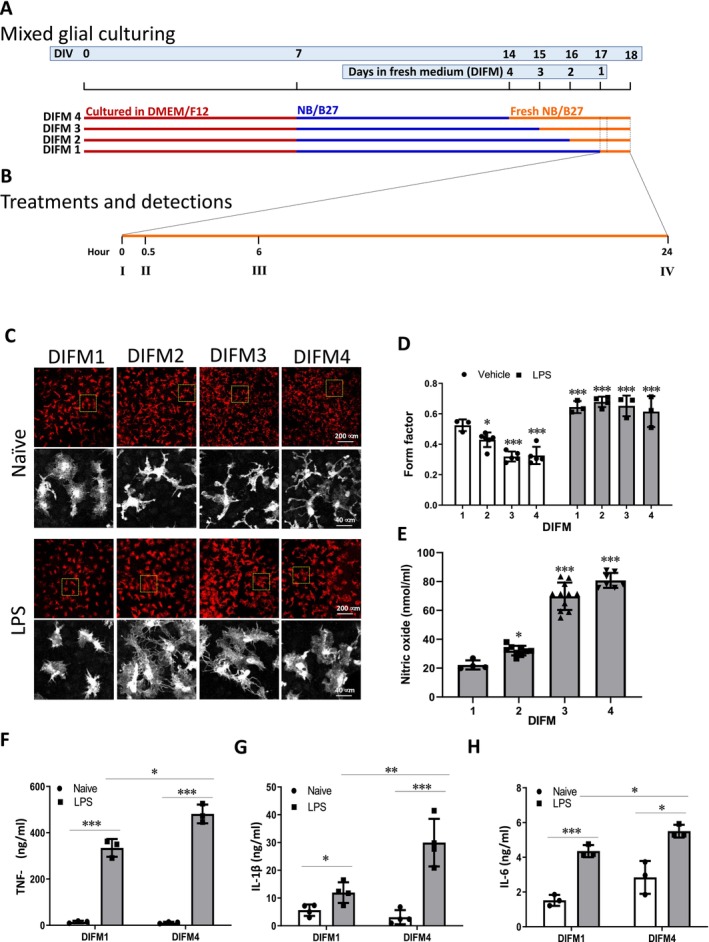
Establishment of mixed glial cultures as cellular models to study microglial morphological and functional phenotypes. (A) Schematic diagram of the culture, maturation, and phenotypic analysis of mixed glial cells. Primary mixed glial cells were prepared from the cerebral cortex of Sprague–Dawley rat pups and maintained in DMEM/F12 medium supplemented with 10% FBS for 7 days (DIV 7). The medium was replaced with Neurobasal Medium with B27 supplement (NB/B27). To detect the effect of medium refreshment, the fresh medium was replaced at DIV 14, 15, 16, and 17 which make the cells were cultured for 4, 3, 2, and 1 days in fresh medium (DIFM), respectively, at DIV18. (B) At DIV17, erinacines and LPS (100 ng/mL) were added with an interval of 0.5 h (I and II), and the detection of nitric oxide, cytokines, and form factor was then performed after DIV 18 (IV). For gene expression analysis, mRNA of the DIFM1 cells were isolated 6 h after the treatment of erinacines or vehicle (III). The mRNAs of the DIFM4 cells were isolated at the same time as DIFM1. (C–H) The cells were immunostained with an Iba‐1 antibody (red), and a representative image is shown (upper panels). The lower panels are magnified images of the upper panels marked with dotted squares (C). The form factor was calculated (D), and the level of nitric oxide was determined (E). TNF‐α (F), IL‐1β (G), and IL‐6 (H) levels in DIFM1 and DIFM4 cells were determined. Results are mean ± SD from three independent experiments. Significant differences between the naïve D1 and other groups (D, E); and the underlined groups are indicated by *p < 0.05, **p < 0.01, and ***p < 0.001 (F–H).

To study the morphological and functional phenotypes of microglia, we established various maturation stages of microglia by culturing mixed glial cells in fresh culture medium for different periods. To access the time‐dependent changes of microglial morphology, the medium was refreshed on the day in vitro (DIV)14, 15, 16, and 17 for DIFM4‐, DIFM3‐, DIFM2‐, and DIFM1‐glia cells, which make the cultures conditioned in fresh medium for 4, 3, 2, and 1 days at DIV18, respectively (Figure 1A). For activation, LPS (100 ng/mL) was added to the cell cultures on DIV17 for 24 h. Morphological changes in microglia and astrocytes were observed at DIV18 (Figure 1B).

Following fixation, the cells were subjected to immunostaining with an anti‐Iba‐1 antibody for microglia and an anti‐GFAP antibody for astrocytes to detect morphological changes (Figures S2; Figure 1C). The results demonstrated a time‐dependent alteration in microglial morphology, with DIFM1‐microglia exhibiting an amoeboid‐like shape, whereas DIFM4‐microglia displayed a ramified morphology. Microglia morphology was quantified using the “form factor.” We found that the form factors of DIFM1‐ and DIFM4‐microglia were 0.55 and 0.35, respectively, which increased to 0.65 when microglia were activated by LPS on DIV17 (Figure 1D). In contrast, no obvious changes in astrocytic morphology were observed during the fresh medium replacement (Figure S2). To assess functional phenotypic changes in LPS‐activated DIFM1 and DIFM4, the levels of proinflammatory factors, including nitric oxide, TNF‐α, IL‐1β, and IL‐6, in the conditioned medium were measured. Compared with DIFM1, DIFM4 increased the nitric oxide, TNF‐α, IL‐1β, and IL‐6 levels by 263%, 43%, 150%, and 26%, respectively (Figure 1E–H). These results suggest that DIFM4‐microglia exhibit greater maturity than DIFM1‐microglia in both morphological and functional phenotypes.

### 3.2 Erinacines Promote Microglial Phenotype Maturation

**FIGURE 2:**
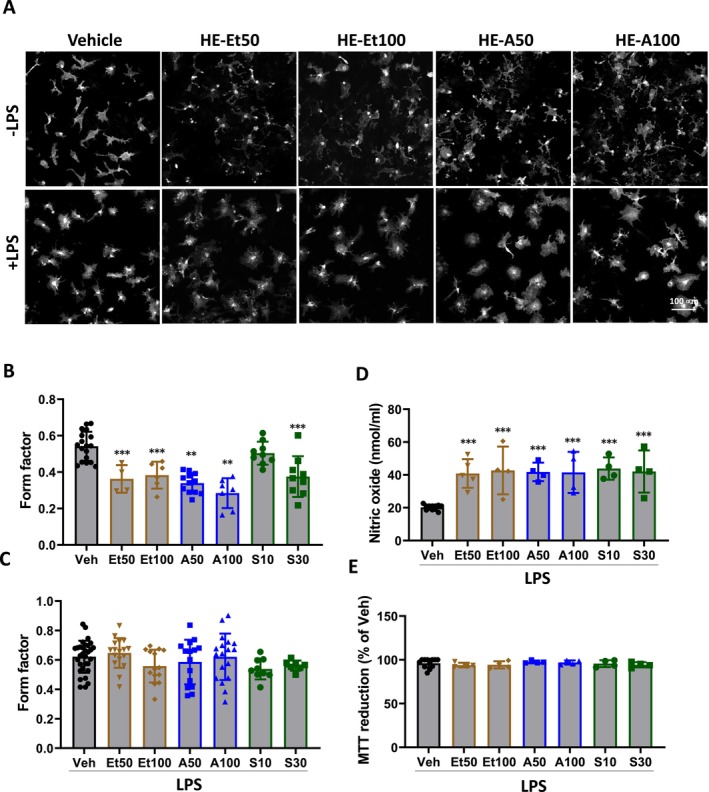
The effect of erinacines on microglial phenotype changes in DIFM1. (A–C) After treatment, cells were fixed and subjected to immunostaining of microglia by anti‐Iba1 antibody. The representative image was shown (A), and the form factor was calculated (B, C). (D, E) After treatment, cells and conditioned medium were collected. Nitric oxide production in the conditioned medium was determined (D). Cell survival was determined by MTT reduction assay (E). Results are mean ± SD from three independent experiments. Significant differences between the cells treated with vehicle (Veh) and the other groups are indicated by **p < 0.01, and ***p < 0.001.

DIFM1 cells contain amoebic microglia and produce low amounts of pro‐inflammatory factors upon LPS stimulation. Therefore, DIFM1 cells were used to study the potential of erinacines to alter microglial morphology and glial phenotype (Figure 1B). Observations revealed that erinacines‐treated DIFM1‐microglia exhibited a ramified morphology and the formation of long protrusions (Figure 2A). The form factor of DIFM1‐microglia is 0.47, which decreases to 0.38, 0.28, and 0.37 after treatment with HE‐Et (100 μg/mL), HE‐A (100 μM), and HE‐S (30 μM), respectively (Figure 2B). Following LPS activation, while no significant difference in form factor was observed (Figure 2A,C), the nitric oxide level (Figure 2D) was significantly increased in DIFM1‐microglia treated with erinacines. In those assays, all erinacines were used at nontoxic concentrations (Figure 2E). Taken together, the effects of erinacines on modulating DIFM1‐microglia into a more ramified morphology and increasing the nitric oxide production in DIFM1 cells after LPS stimulation indicate that erinacines promote the transformation of microglia from an immature to a more mature phenotype.

### 3.3 HE‐A Promotes the Polarization of Microglia Toward the Anti‐Inflammatory M2‐Like Phenotype

**FIGURE 3:**
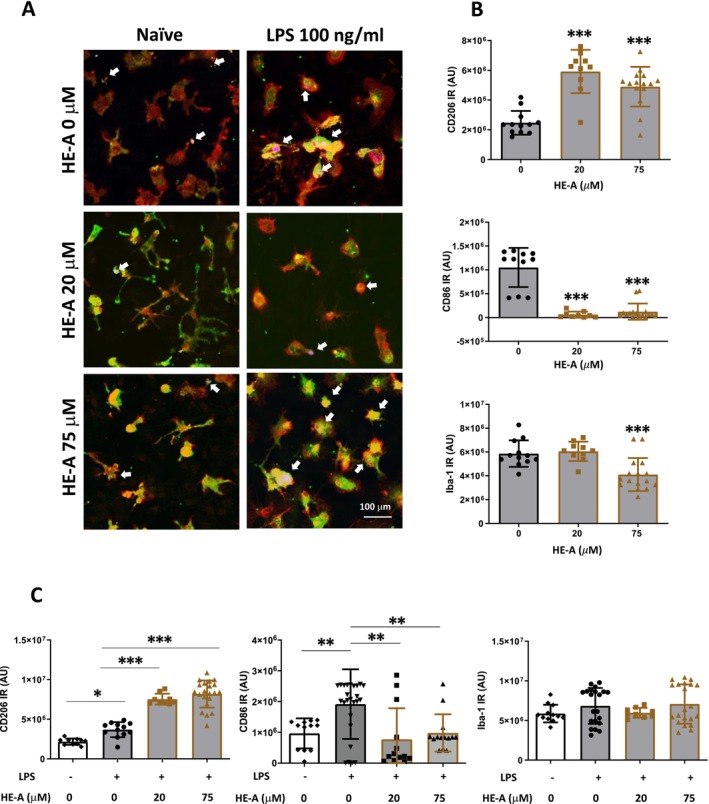
The effect of HE‐A on M1/M2 polarization of DIFM1‐microglia. The cells were cultured and activated as depicted in Figure 1A. After treatment, cells were fixed and subjected to immunostaining of microglia by anti‐Iba‐1 antibody (red), M2 marker by anti‐CD206 antibody (green), and M1 marker by anti‐CD86 antibody (blue). A. The representative image was shown. B. The immunoreactivity (IR) of CD206 (left panel), CD86 (middle panels), and Iba‐1 (right panels) of the microglia treated with HE‐A (B) and HE‐A plus LPS (C) were determined using ImageJ software. Results are mean ± SD from three independent experiments. Significant differences between the HE‐A group and the other groups are indicated by *p < 0.05, **p < 0.01 and ***p < 0.001.

Next, we investigated the effect of HE‐A on the DIFM1‐microglia phenotype. DIFM1 cells were treated with HE‐A alone or in combination with LPS (100 ng/μl). After 24 h of treatment, immunostaining was conducted to distinguish M1 and M2 microglia using CD86 and CD206 antibodies, respectively. Compared with the control group, HE‐A treatment significantly increased CD206 expression and decreased CD86 expression in DIFM1‐microglia (Figure 3A,B). expression. Compared with treatment with LPS alone, treatment with HE‐A plus LPS increased CD206 expression in a concentration‐dependent manner, while concurrently reducing CD86 (Figure 3A,C). These findings suggest that HE‐A promotes the differentiation of microglia into an anti‐inflammatory M2‐like phenotype, suggesting that erinacines promote microglia toward neuroprotective phenotype.

### 3.4 CMap Analysis Uncovers Potential Mechanisms of Erinacines in Modulating Microglia Phenotype, Highlighting HDAC Inhibition

To elucidate the potential pathways targeted by erinacines, we conducted a Connectivity Map (CMap) analysis to compare the gene expression profiles of groups DIFM1 (D1), DIFM4 (D4), HE‐A‐treated DIFM1 (A1), HE‐C‐treated DIFM1 (C1), and HE‐S‐treated DIFM1 (S1), with group D1 serving as the reference. This analysis encompassed “perturbagen classes” (PCLs)—categorized by mechanism of action—and “perturbagen compounds” (PCs). The “score” metric quantifies the similarity between the treatment effects and the corresponding PCL or PC, ranging from 100 (identical) to −100 (opposite). This score can indicate genes targeted or affected by drug treatment. For the analysis, varying concentrations of HE‐A (10 μM), HE‐S (10 and 30 μM), and HE‐C (10 and 30 μM) were applied to each erinacine group.

CMap analysis identified PCLs with scores exceeding 80.0 for groups D4, A1, S1, and C1, totaling 10, 1, 9, and 14, respectively (Table 1). Within group D4, the three predominant PCLs were bromodomain inhibitors, leucine‐rich repeat kinase inhibitors, and histone deacetylase (HDAC) inhibitors. Notably, HDAC inhibitors were identified as a shared PCL across groups D4, S1, and C1. Additionally, heat shock protein (HSP) inhibitors were common in groups D4 and C1.

**TABLE 1:** CMap result: Perturbagen class (PCL) of the four groups.

PCL	Score	Common group
D4 group		
Bromodomain inhibitor	96.44	
Leucine‐rich repeat kinase inhibitor	95.59	
HDAC inhibitor	95.34	S1, C1
Topoisomerase inhibitor	91.09	
PI3K inhibitor	89.36	
MEK inhibitor	88.85	
SRC inhibitor	87.13	
Minor histocompatibility	85.11	
HSP inhibitor	83.51	C1
General transcription factors group 2 LOF	80.25	
A1 group		
MTOR inhibitor	90.22	
S1 group		
Vesicular transport LOF	98.00	C1
Proteasome inhibitor	97.37	C1
Heat shock 70 kDa proteins LOF	96.55	
HIF activator	93.31	C1
HIV protease inhibitor	91.09	C1
HDAC inhibitor	86.58	D4; C1
Protein phosphatase catalytic subunits LOF	86.34	
Wnt family GOF	84.05	C1
EIF Proteins LOF	81.50	C1
C1 group		
Wnt family GOF	96.79	S1
BCL inhibitor	96.24	
HDAC inhibitor	95.11	D4; S1
HIV protease inhibitor	94.78	S1
PKC activator	93.67	
PKC inhibitor	93.28	
Vesicular transport LOF	93.12	S1
HSP inhibitor	87.43	D4
EIF Proteins LOF	87.11	S1
IKK inhibitor	85.91	
X linked mental retardation group 2 LOF	85.79	
HIF activator	85.12	S1
Proteasome inhibitor	82.82	S1
EGFR inhibitor	80.10	

*Note:* The PCL, perturbagen score, and common functions to other groups were indicated. The PCL of the D4 group common to S1, and C1 groups were indicated.

Abbreviations: BCL, B‐cell leukemia/lymphoma‐2; DIFM, days in fresh medium; EGFR, epidermal growth factor receptor; EIF, eukaryotic initiation factor; GOF, gain‐of‐function; HDAC, histone deacetylase; HIF, hypoxia‐inducible factor; HIV, human immunodeficiency virus; IKK, IκB kinase; LOF, loss‐of‐function; MEK, mitogen‐activated protein kinase; MTOR, mammalian target of rapamycin; PCL, perturbagen class; PI3K, phosphoinositide 3‐kinase; PKC, protein kinase C; SRC, Src tyrosine kinase.

**FIGURE 4:**
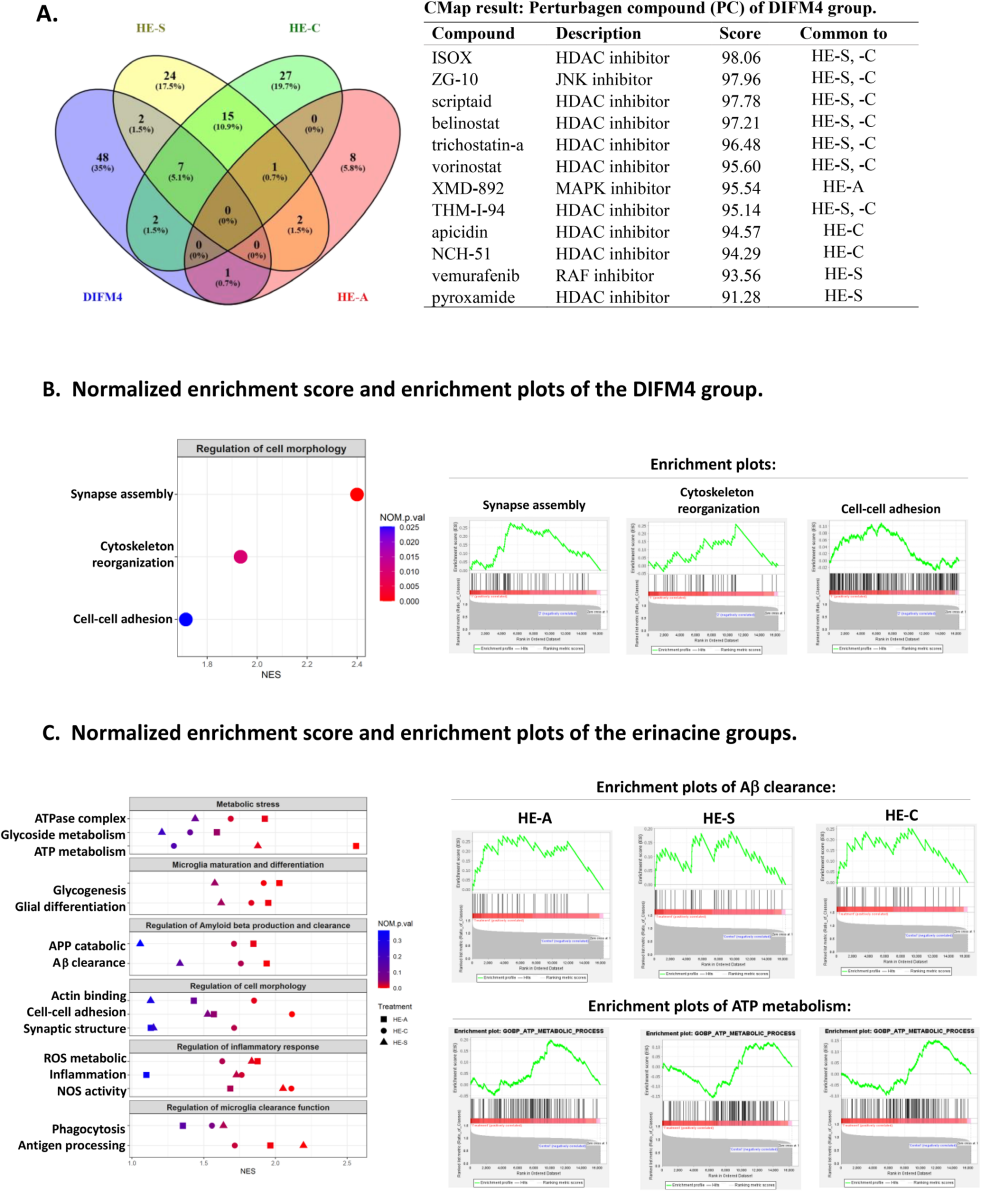
Gene analysis data of DIFM4 and DIFM1 treated with erinacines. (A) The L1000 gene expression profiles of the D4, A1, S1, and C1 groups compared with those of the D1 group were analyzed via CLUE. The PCs (score > 80) of the four groups were intersected and are shown as a Venn diagram. The connectivity score is based on the Kolmogorov–Smirnov enrichment statistical evaluation of each gene expression profile. The results provided from CLUE are expressed as a comprehensive connectivity score, showing that the same drug has a similar MOA on different groups in CLUE. The PCs of the D4 group common to the PCs of erinacines are shown in the right panel. B, C. Gene ontology (GO) term enrichment analysis by GSEA in detected comparisons. (B) Significant upregulated GO terms enriched in GSEA analysis for D4 compared to D1. Treatment types are indicated by shape, and *p*‐values are indicated by point colors. Enrichment plots of upregulated regulation of cell morphology in D4 compared to D1. (C) Significant upregulated GO terms enriched in GSEA analysis for erinacine treatments compared to D1. Treatment types are indicated by shape, and *p*‐values are indicated by point colors. Enrichment plots of upregulated amyloid‐beta clearance in erinacine treatments. Aβ, amyloid β protein; GO, gene ontology; GOBP, gene ontology biological process; GOCC, gene ontology cellular component; GOMF, gene ontology molecular function; GSEA, Gene Set Enrichment Analysis; HDAC, histone deacetylase; JNK, c‐Jun N‐terminal kinase; MAPK, mitogen‐activated protein kinase; NES, normalized enrichment scores; NOM *p* val, normalized *p*‐value; NOS, nitric oxide synthase; PC, perturbagen compound; ROS, reactive oxygen species.

For perturbagen compounds (PCs) with scores above 90.0, the D4 group matched with 60 PCs, the A1 group with 11 PCs, the S1 group with 51 PCs, and the C1 group with 52 PCs, as indicated in Table S1 and other data not shown. Upon further analysis of the intersecting results from these groups, seven PCs were found to have shared functions among groups D4, C1, and S1 (Figure 4A; Table S1). Specifically, two PCs, XMD‐885 (a mitogen‐activated protein kinase inhibitor) and XMD‐892 (an AMPK inhibitor), were common between groups D4 and A1. Additionally, two PCs, apicidin and NCH‐51 (the HDAC inhibitors), were shared between groups D4 and C1. Vemurafenib and pyroxamide, which act as an RAF kinase inhibitor and an HDAC inhibitor, were common between groups D4 and S1 (Figure 4A; Table S1). Furthermore, one PC, WT‐171 (an HDAC inhibitor), was common among groups A1, C1, and S1. This consistent association with HDAC inhibitors across multiple groups, combined with their similar mechanisms of action, suggests that erinacines may exert their effects, at least in part, by modulating HDAC activity.

### 3.5 Insights From GSEA: Erinacines Upregulate Pathways for Microglial Maturation, Metabolic Function, and Amyloid‐Beta Clearance

We utilized Gene Set Enrichment Analysis (GSEA) to investigate significant differences in gene expression across the phenotypes of groups D4, A1, C1, and S1. A positive Normalized Enrichment Score (NES) indicates upregulated gene enrichment. Our findings revealed that relative to group D1, group D4 exhibited significant enrichment in biological processes pivotal for morphological transformation, such as synapse assembly regulation (NES = 2.35, *p* = 0.0016), plasma membrane‐mediated cell–cell adhesion (NES = 1.7, *p* = 0.02), and cytoskeleton reorganization (NES = 1.9, *p* = 0.009), as depicted in Figure 4B. Additionally, the enrichment plots further illustrate the regulation of cell morphology.

Significantly, the erinacine‐treated groups displayed upregulation of genes associated with key processes influencing microglial morphology, including cell–cell adhesion regulation (NES = 1.57, *p* = 0.04 for S1; NES = 2.1, *p* = 0.001 for C1; NES = 1.68, *p* = 0.03 for A1), synaptic structure modification (NES = 1.7, *p* = 0.01 for group C1), and actin filament binding (NES = 1.85, *p* = 0.01 for group C1), suggesting their influence on microglial morphology. Moreover, the role of erinacines, particularly HE‐A, in promoting microglial maturation is further supported by the enhancement of gliogenesis (NES = 2.02, *p* = 0.002 for group A1; NES = 1.91, *p* = 0.004 for group C1; NES = 1.57, *p* = 0.04 for group S1), glial cell differentiation (NES = 1.95, *p* = 0.005 for group A1; NES = 1.83, *p* = 0.01 for group C1; NES = 1.62, *p* = 0.039 for group S1), nitric oxide synthase activity (NES = 1.68, *p* = 0.03 for group A1; NES = 2.1, *p* = 0.003 for group C1; NES = 2.05, *p* = 0.005 for group S1), microglial phagocytosis capabilities including antigen processing and presentation (NES = 1.68, *p* = 0.02 for group A1; NES = 2.19, *p* = 0.001 for group S1), and phagocytosis (NES = 1.63, *p* = 0.03 for group S1), as shown in Figure 4C. These findings highlight the potential of erinacines to modulate microglial morphology and function, promoting a transition toward a more mature and neuroprotective phenotype.

Furthermore, erinacine treatments, particularly with HE‐A, significantly enriched pathways related to energy metabolism and amyloid‐beta clearance. Specifically, we observed enrichment in the ATPase complex (NES = 1.9, *p* = 0.009 for group A1; NES = 1.6, *p* = 0.01 for group C1), glycoside metabolic processes (NES = 1.59, *p* = 0.04 for group A1), and ATP metabolic processes (NES = 2.56, *p* < 0.001 for group A1). Additionally, treatments with HE‐A and HE‐C were notably enriched in pathways associated with Aβ clearance (NES = 1.93, *p* = 0.003 for group A1; NES = 1.76, *p* = 0.03 for group C1) and the regulation of the amyloid precursor protein catabolic process (NES = 1.84, *p* = 0.009 for group A1; NES = 1.71, *p* = 0.02 for group S1), as illustrated in Figure 4C. Given that impaired energy metabolism is a hallmark of AD, these results suggest that erinacines may influence both energy metabolism and Aβ clearance, which are critical factors in the progression of AD. These findings raise the possibility that erinacines could be particularly beneficial in addressing the metabolic challenges commonly seen in AD patients.

### 3.6 Commonly Regulated Genes and Biological Processes Across Erinacine‐Treated Groups

**FIGURE 5:**
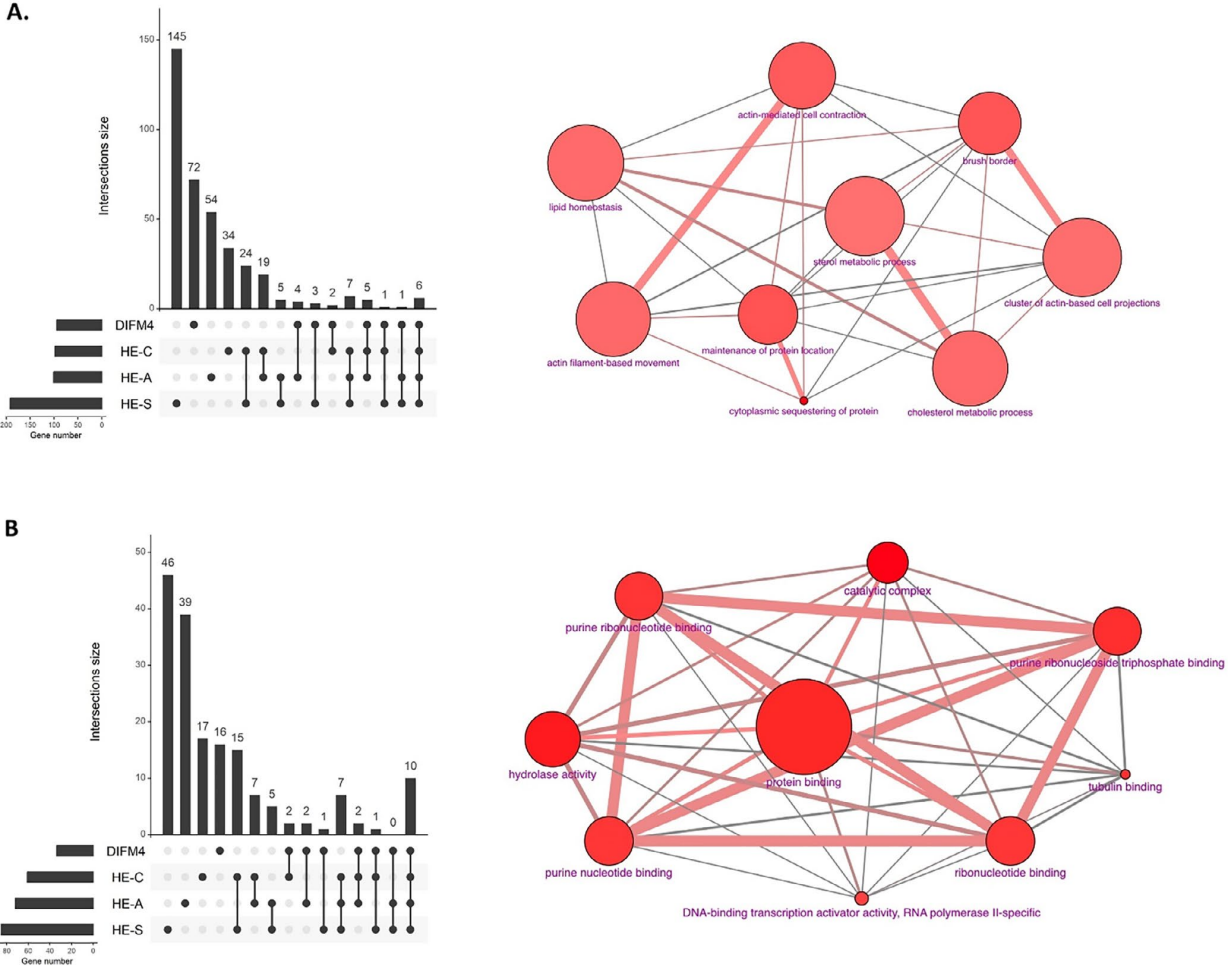
The upregulated gene expression and downregulated gene expression of DIFM4 and DIFM1 treated with erinacines. The upregulated gene expression and downregulated gene expression of the four groups were intersected by R programming. The significantly enriched biological processes of the common upregulated genes (expression ratio ≥ 2) (A) and downregulated genes (expression ratio ≤ −2) (B) of the four groups were analyzed via CPDB to reveal the functions of the genes and construct the network view. The size of nodes and lines is proportional to the number of members and the overlap of the gene numbers. Each node represents the number of genes or metabolites contained, and the *p*‐value is encoded as the node size and node color, respectively. Two nodes are connected by an edge if they share members. The edge width reflects the relative overlap between the nodes, while the edge color encodes the number of the same shared members that are also found in the user's input gene lists. CLUE, Connectivity Map, and Library of Integrated Network‐Based Cellular Signatures (LINCS) unified environment; CPDB, ConsensusPathDB.

To identify key genes involved in modulating glial phenotype, we analyzed the differential expression of genes (with an expression ratio of ≥ 2 or ≤ −2) among groups D4, A1, S1, and C1. Group D4 exhibited a twofold upregulation in 96 genes, as listed in Table S2. Notably, six genes (Rbp1, Tpm2, sf3a3, Capn12, Ncapd3, and BC024139) were shared across all three erinacine‐treated groups, as shown in Figure 5A and Table 2. Additionally, five genes (R1f, Dab1, pmm1, Nrg2, and Ogfrl1) were common between groups A1 and C1; Pla2g15 was shared by groups A1 and S1; Dzip1 was shared by groups C1 and S1; while Sri, Atpla2, Tnks2, and Slc13a3 were unique to group A1; Nudt21 and Lima 1 to group C1; and Hdlbp, Parkar1b, and Thbs1 to group S1. The most significantly enriched biological processes among the commonly upregulated genes across the four groups were cytoplasmic sequestering of protein (GO:0051220), maintenance of protein location (GO:0045185), brush border formation (GO:0005903), actin‐mediated cell contraction (GO:0070252), actin filament‐based movement (GO:0030048), cholesterol metabolism (GO:0008203), lipid homeostasis (GO:0055088), clustering of actin‐based cell projections (GO:0098862), and sterol metabolism (GO:0016125), as detailed in Figure 5A and Table S4.

**TABLE 2:** Common upregulated genes in D4 and D1‐treated erinacines.

Gene	Description	Fold	Common group
Thbs1	Platelet aggregation, angiogenesis, and tumorigenesis	2.75	S1
Rlf	DNA hypomethylation, epigenetic gene silencing	2.74	A1, C1
Tnks2	Inhibits TERF1 bind to telomeric DNA	2.7	A1
Rbp1	Transport of retinol from the liver to peripheral tissue	2.67	A1, S1, C1
Nudt21	3’ RNA cleavage and polyadenylation processing	2.63	C1
Tpm2	Critical to normal cardiac function	2.59	A1, S1, C1
Sri	Response to cellular stress in neurodegeneration	2.38	A1
Dab1	The migration and differentiation of neurons	2.35	A1, C1
Pmm1	GDP‐mannose synthesis	2.25	A1, C1
Sf3a3	Subunit 3 of the splicing factor 3a protein complex	2.25	A1, S1, C1
Dzip1	Required for ciliogenesis in cultured mammalian cells	2.22	C1, S1
Nrg2	Induces the growth and differentiation of cells	2.19	A1, C1
Capn12	Participants in cell mobility and cell cycle progression	2.17	A1, S1, C1
Prkar1b	A regulatory subunit of PKA	2.15	S1
Atp1a2	Maintaining the electrochemical gradients of Na^+^/K^+^	2.12	A1
Hdlbp	The regulation of gene expression	2.12	S1
Slc13a3	Transport Krebs cycle intermediates	2.12	A1
Ncapd3	Chromosome assembly and segregation	2.12	A1, S1, C1
Lima1	Embryonic development and cell lineage determination	2.07	C1
Pla2g15	Regulate the multifunctional lysophospholipids	2.04	A1, S1
Ogfrl1	Important in embryonic development and wound repair	2.03	A1, C1
BC024139	Regulation of focal adhesion assembly	2.02	A1, S1, C1

Abbreviations: DIFM, days in fresh medium; PKA, protein kinase A; TERF1, telomeric repeat binding factor 1.

In group D4, we observed a twofold downregulation of 32 genes (Table S3). Among these genes, 10 genes (Boc, Carl3, Cyp4a31, Dcaf12l1, Gna14, Igbo‐V7183, Plpp6, Rio1, Spcs1, and Trip13) were consistently downregulated across all three erinacine groups (Figure 5B and Table 3). Additionally, Ndn and Ube2z were common to groups A1 and C1; Mapre1 and Irf1 to group A1; Tfap2a to group S1; and Zbtb16 and Gm38396 to group C1. Notably, Cx3cr1 was not downregulated in any erinacine group, which may suggest its role in maintaining microglial quiescence. The biological processes enriched among the commonly downregulated genes included catalytic complex (GO:1902494), hydrolase activity (GO:0016787), various forms of nucleotide binding (GO:0035639, GO:0032555, GO:0017076, and GO:0032553), protein binding (GO:005515), tubulin binding (GO:0015631), and DNA‐binding transcription activator activity specific to RNA polymerase II (GO:0001228), as depicted in Figure 5B and Table S4.

**TABLE 3:** Common downregulated genes in D4 and D1‐treated erinacines.

Gene	Description	Fold	Common Group
Zbtb16	Involved in cell cycle progression	−3.8	S1
Spcs1	Has peptidase activity and ribosome binding activity	−2.69	A1, S1, C1
Igh‐V7183	Large polypeptide subunit of an antibody	−2.65	A1, S1, C1
Riok1	Essential in the maturation of 40S subunits	−2.36	A1, S1, C1
Calr3	Binds to misfolded proteins and prevents their export	−2.34	A1, S1, C1
Irf1	Transcription factor for the expression of IFN‐β	−2.26	A1
Gna14	Activates phospholipase C‐β (PLC‐β)	−2.26	A1, S1, C1
Trip13	Hormone‐dependent transcription factors	−2.22	A1, S1, C1
Ndn	Suppresses growth in postmitotic neurons	−2.14	A1, C1
Plpp6	Involved in phospholipid dephosphorylation	−2.12	A1, S1, C1
Boc	Participates in axon guidance and the smoothened signaling pathway	−2.11	A1, S1, C1
Ube2z	Facilitates protein ubiquitination for degradation machinery	−2.11	A1, C1
Mapre1	A microtubule plus end tracking protein	−2.07	A1
Gm38396	Sphingomyelin phosphodiesterase	−2.06	C1
Cyp4a31	Functions as a fatty acid dehydrogenase and monooxygenase	−2.04	A1, S1, C1
Dcaf12l1	Function in cell cycle, apoptosis, and gene regulation	−2.03	A1, S1, C1
Tfap2a	Enhances transcription by binding to a GC‐rich sequences	−2.02	S1

Abbreviations: DIFM, days in fresh medium; IFN‐β, interferon‐β; PLC‐β, phospholipase C‐β.

### 3.7 HDAC Inhibitors Promote Microglial Phenotype Change in DIFM1‐Microglia

Based on the CMap result, erinacines appear to modulate microglial maturation through HDAC inhibition. To confirm the involvement of HDAC in microglial phenotype changes, amoeboid DIFM1‐microglia were treated with HDAC inhibitors at DIV17. Two acridine‐based HDAC inhibitors, designated as 11b and 11 h, were utilized in this study. Compound 11b demonstrates superior inhibitory activity against class IIa and class IIb HDACs compared to suberoylanilide hydroxamic acid (SAHA), while 11 h selectively inhibits HDAC6 [39]. Additionally, 11b has been identified as a multitarget inhibitor that affects HDAC, Aβ aggregation, and acetylcholine esterase (AChE).

**FIGURE 6:**
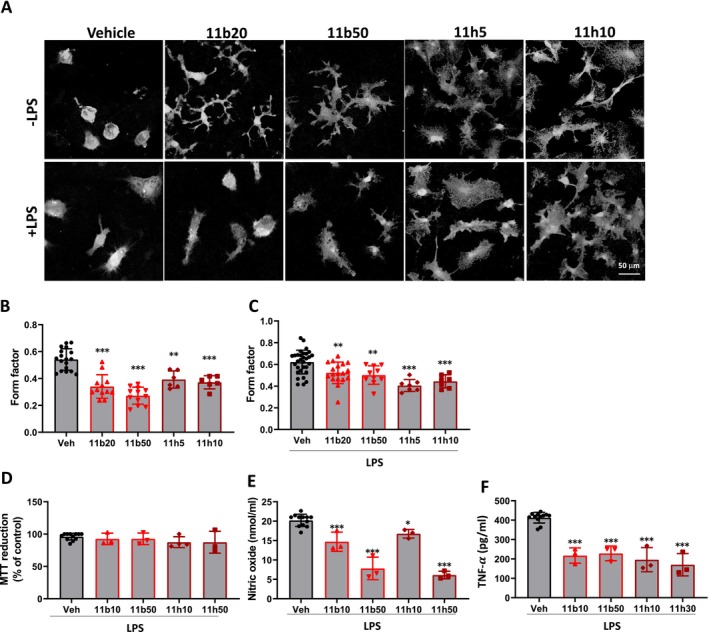
The effect of HDAC inhibitors on microglial phenotype changes in DIFM1. (A–C) After treatment, cells were fixed and subjected to immunostaining of microglia with an anti‐Iba1 antibody. The representative image was shown (A), and the form factor was calculated (B, C). (D–F) After treatment, cells and the conditioned medium were collected. Cell survival was determined by the MTT reduction assay (D). Nitric oxide production (E) and the expression of TNF‐α (F) were determined. The results are presented as the means ± SD from three independent experiments. Significant differences between the vehicle (B–F) and the other groups are indicated by *p < 0.05, **p < 0.01, and ***p < 0.001.

Following HDAC inhibitor treatment, DIFM1‐microglia were activated with LPS (100 ng/μl) as depicted in Figure 1. After 24 h, cells were harvested and subjected to immunostaining with an anti‐Iba‐1 antibody to assess microglial morphology. Remarkably, DIFM1‐microglia treated with HDAC inhibitors 11b and 11 h exhibited ramification and developed elongated protrusions, indicating a transition to a surveillance state (Figure S3). The form factor of microglia decreased from 0.52 in the control group to 0.27 and 0.37 in the groups treated with 11b (50 μM) and 11 h (10 μM), respectively (Figure 6A,B). Notably, even after LPS activation, 11b and 11 h continued to reduce the form factor of DIFM1‐microglia from 0.62 in the control to 0.50 and 0.44 in the 11b (50 μM) and 11 h (10 μM)‐treated groups, respectively (Figure 6A,C). Additionally, at nontoxic concentrations (Figure 6D), there was a significant decrease in the levels of nitric oxide and TNF‐α following treatment with either 11b or 11 h (Figure 6E,F). These observations suggest that HDAC inhibitors can promote microglia toward a ramified, surveillance‐like state, and suppress microglial activation. This supports our hypothesis that erinacines might target HDAC activity to promote microglia ramification; however, erinacines might not specifically target HDACs for functional phenotypic changes.

### 3.8 Impact of Erinacines on Glycemic Control in HFSTZ‐APP/PS1 Mice

**FIGURE 7:**
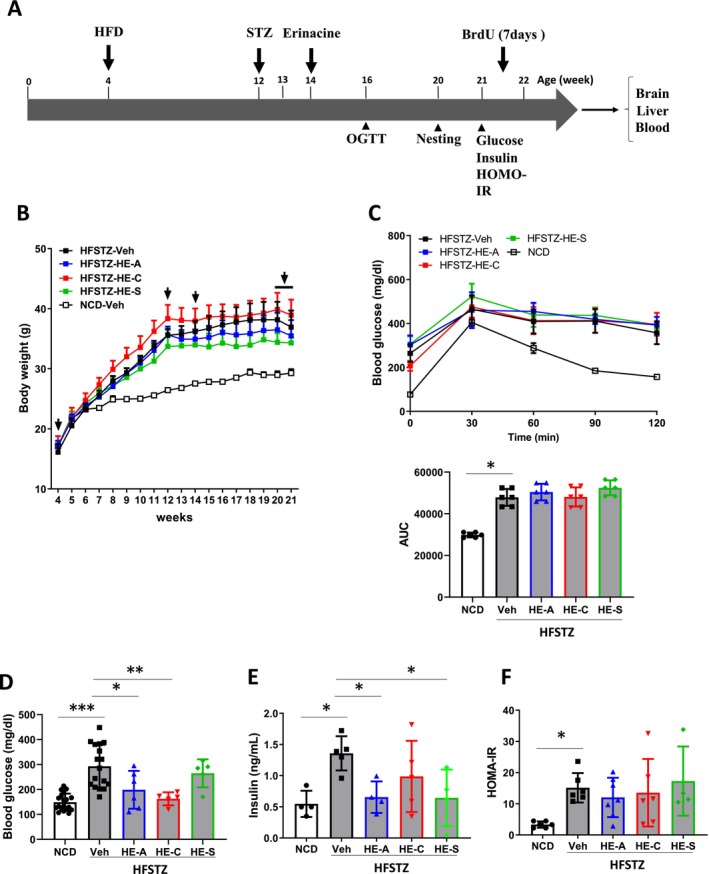
Experimental design and impact of erinacines on glycemic control. (A) APP/PS1 mice were fed a high‐fat diet (HFD) at 4 weeks of age. At 12 weeks of age, low‐dose streptozotocin (STZ) was administered intraperitoneally. At 14 weeks of age, administration of vehicle (Veh) and HE‐A (30 mg/kg), HE‐C (30 mg/kg), or HE‐S (30 mg/kg) was started (*n* = 6 for each group). The nesting behavior ability test was conducted at 20 weeks of age, and finally, the mice were sacrificed after continuous BrdU administration for 7 days. (B) Body weight was measured weekly after 4 weeks of age. (C) The results of the oral glucose tolerance test (OGTT) of HFSTZ mice at 16 weeks of age (upper panel), the results, and the quantification of their area under the curve (AUC) mean (lower panel). (D) Fasting blood glucose (E) fasting insulin and (F) HOMA‐IR were determined at 21 weeks old. The results are expressed as mean ± SEM, and the comparison between each group and the normal chow diet (NCD) group is expressed as *p < 0.05; **p < 0.01, and ***p < 0.001. BrdU, 5‐bromo‐2′‐deoxyuridine; HFSTZ, HFD plus STZ; HOMO‐IR, Homeostasis Model Assessment‐Insulin Resistance.

Given that our GSEA indicated that erinacines upregulate metabolic regulation and Aβ clearance pathways, we hypothesized that erinacines could be beneficial for treating AD under conditions of metabolic stress. To test this hypothesis, we examined the effects of erinacines on HFSTZ‐APP/PS1 mice (*n* = 6 for each group). The experimental protocol began by placing the mice on a high‐fat diet (HFD) at 4 weeks of age. After 8 weeks on HFD (at 12 weeks of age), the mice received an intraperitoneal injection of STZ (50 mg/kg). Starting at 14 weeks of age, the mice were administered with erinacines at a dosage of 30 mg/kg/day. An oral glucose tolerance test was performed at 16 weeks, followed by an evaluation of nesting behavior at 20 weeks. The mice were sacrificed after a continuous 7‐day administration of BrdU, and samples of their brains, serum, and livers were collected (Figure 7A).

At 12 weeks, the HFD successfully increased the body weight of the mice to 36.65 g, whereas the body weight of the mice fed a normal chow diet (NCD) was 26.45 g (Figure 7B). Post‐STZ administration, the mice stopped gaining weight and displayed elevated fasting blood glucose levels at 16 and 21 weeks, indicating successful induction of metabolic stress in APP/PS1 mice (Figure 7C,D). The oral glucose tolerance test at 16 weeks revealed that, unlike NCD mice, HFSTZ mice's blood glucose levels did not return to normal after glucose administration (Figure 7C). Treatment with erinacines did not result in significant changes in body weight or oral glucose tolerance compared to the control group. However, erinacine treatments, specifically HE‐A and HE‐S, significantly reduced fasting blood glucose and insulin levels (Figure 7E). Nonetheless, the HOMA‐IR remained unchanged across all erinacine treatments (Figure 7F), suggesting that erinacines exert a limited effect on metabolic stress in HFSTZ mice.

### 3.9 Erinacines Improve the Nesting Behavior of HFSTZ‐APP/PS1 Mice

**FIGURE 8:**
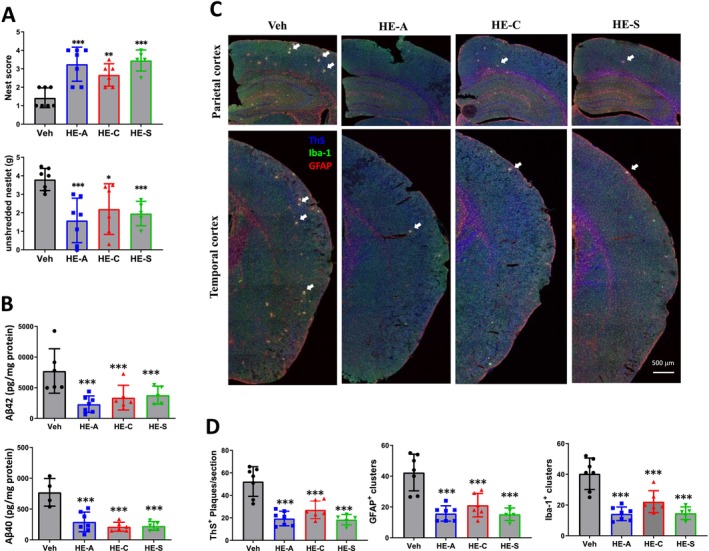
Erinacines improve the pathologies in HFSTZ‐APP/PS1 mice. HFSTZ‐APP/PS1 mice were fed with vehicle (Veh), HE‐A (30 mg/kg), HE‐C (30 mg/kg), or HE‐S (30 mg/kg) for 6 weeks (*n* = 6 for each group). (A) Testing of nesting behavior ability was detected at 21 weeks old. Nest scores and the weight of unshredded nestlets were shown. The amount of insoluble Aβ in the cerebral homogenate was detected (B). (C, D) Mouse brains were sliced for tissue immunofluorescent staining. Aβ plaques were detected using Thioflavin S (blue), astrocytes and microglia were detected using GFAP antibodies (red) and Iba‐1 antibodies (green), respectively, and the representative images are shown in panel C. Statistical diagram of the number of Aβ plaques and the number of glial cell clusters is shown in panel D. The results are expressed as mean ± SEM, and the comparisons between each group and the Veh group are expressed as *p < 0.05, **p < 0.01 and ***p < 0.001. Aβ, amyloid β protein; GFAP, glial fibrillary acidic protein; Iba‐1, ionized calcium‐binding adaptor molecule 1.

To assess the effects of erinacines on the daily behavior of HFSTZ mice, we administered HE‐A, HE‐C, and HE‐S at a dosage of 30 mg/kg/day. Six weeks post‐administration, we conducted a nesting behavior task to evaluate the structural integrity of the nests (nest score) and to measure the weight of the remaining unshredded nestlets (Figure 8A). The nest scores for the vehicle, HE‐A, HE‐C, and HE‐S groups were 1.429 ± 0.202, 3.25 ± 0.3493, 2.667 ± 0.2472, and 3.45 ± 0.255, respectively. Correspondingly, the weights of the unshreded nestlets were 3.8 ± 0.2257 g, 1.586 ± 0.4543 g, 2.2 ± 0.558 g, and 1.96 ± 0.2943 g. These results indicated that erinacine treatments can improve memory and behavioral deficits in HFSTZ‐APP/PS1 mice.

### 3.10 Erinacines Reduce Aβ Plaques and Plaque‐Associated Glial Activation in the Brains of HFSTZ‐APP/PS1 Mice

Following the administration of erinacines to HFSTZ‐APP/PS1 mice, the animals were euthanized, and the levels of Aβ in the cerebral cortex were quantified via ELISA analysis. The findings revealed that erinacine treatments significantly reduced the levels of SDS‐insoluble Aβ1‐42 and Aβ1‐40 (Figure 8B), while the levels of SDS‐soluble Aβ remained unaffected (data not shown). The brain sections were then analyzed with Thioflavin S (ThS) staining to visualize amyloid plaques and immunostaining with GFAP and Iba‐1 antibodies to identify astrocytes and microglia, respectively. The results demonstrated that glial cells clustered around amyloid plaques (Figure 8C). Quantitative analysis revealed the number of ThS‐positive plaques in the vehicle, HE‐A, HE‐C, and HE‐S treated groups as 52.29 ± 4.99, 19.43 ± 2.45, 27.08 ± 3.20, and 18.4 ± 2.05, respectively (Figure 8D). The numbers of GFAP‐positive clusters were 42.36 ± 4.50, 15.79 ± 1.88, 21.17 ± 3.10, and 15.3 ± 1.83 across the four groups, while the numbers of Iba‐1‐positive clusters were 40.36 ± 3.87, 14.29 ± 1.69, 22.17 ± 2.92, and 14.70 ± 1.81 for the same group.

**FIGURE 9:**
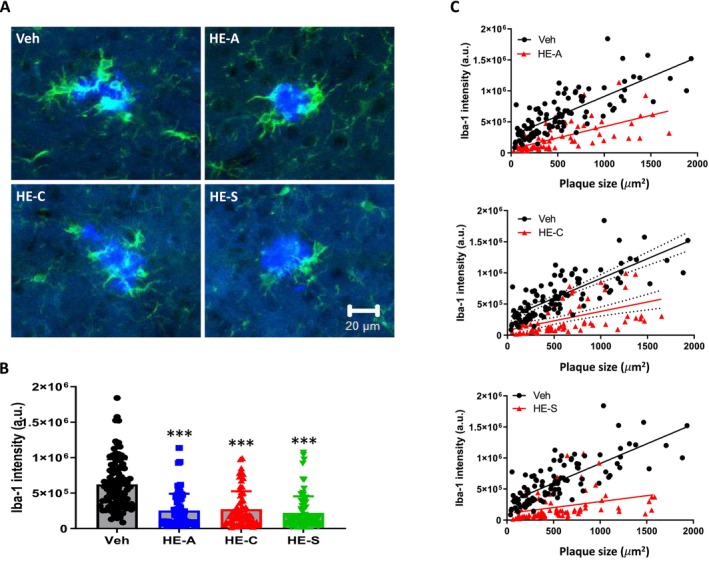
Erinacines reduce plaque‐associated microglial activation in the brains of HFSTZ‐APP/PS1 mice. HFSTZ‐APP/PS1 mice were fed with vehicle (Veh), HE‐A (30 mg/kg), HE‐C (30 mg/kg), or HE‐S (30 mg/kg) for 6 weeks (*n* = 6 for each group), and brain sections were taken for tissue immunofluorescence. Aβ plaques were detected using Amylo Glo (blue), and microglia were detected using Iba1 antibody (green). In total, 15 to 20 plaques were taken from each mouse brain section to observe the degree of activation of the surrounding microglia. Representative image of microglial activation around plaques (A). Quantification of fluorescence intensity of Iba1 signals around the plaque of different drug and Veh groups (B). The results are expressed as mean ± SEM. The comparison between each group and the Veh group is represented by *p < 0.05, **p < 0.01, and ***p < 0.001. (C) Scatter diagrams of the plaque area and Iba1 signal fluorescence intensity around the plaque for different drug and Veh groups. (The solid line represents linear regression; the dotted line represents the 95% confidence interval). HFSTZ‐APP/PS1 mice were fed with vehicle (Veh), HE‐A (30 mg/kg), HE‐C (30 mg/kg), or HE‐S (30 mg/kg) (*n* = 6 for each group) and were injected with BrdU (50 mg/kg) for seven consecutive days before sacrifice, and brain sections were taken for tissue immunofluorescence staining. BrdU antibody was used to detect Type 2 neural progenitor (green), and Doublecortin (DCX) antibody was used to detect newborn neurons (red). Representative immunofluorescence staining of newborn neurons in the SGZ in the hippocampus (A). The arrows indicate BrdU^+^ Type 2 neural progenitor, the open arrows indicate DCX^+^ new neurons, and the double arrows indicate BrdU^+^ DCX^+^ newborn neurons that have just completed differentiation. (B, C) Quantification of BrdU^+^ cells/mm (B) and DCX^+^ cells/mm (C) in the SGZ. The dendritic complexity was analyzed by laminar quantification (D). (E, F) Primary (pri.) and secondary (sec.) dendrites of the DCX^+^ cells are counted along the middle of GCL (green dashed line) and the outer edge of GCL (red dashed line), respectively. The sprouting ratio of the primary dendrites and the branching ratio of the DCX^+^ cells were shown. The results are presented as the mean ± SEM. Significant differences between the Veh group and the other groups are indicated by ***p < 0.001. ML, molecular layer; GCL, granular cell layer; SGZ, subgranular zone.

To further elucidate the interaction between amyloid plaques and microglia, brain sections were stained with Amylo Glo for amyloid plaque detection and with an Iba‐1 antibody for microglia. The fluorescence intensity of both Amylo Glo and the Iba‐1 signal proximal to the plaques was quantified using Metamorph software (Figure 9A). The fluorescence intensity values for Iba‐1 in the vehicle, HE‐A, HE‐C, and HE‐S groups were 621,901 ± 34,638, 254,083 ± 29,067, 272,017 ± 30,074, and 220,618 ± 25,463, respectively (Figure 9B). Compared to the vehicle group, a notable reduction in the Iba‐1 signal intensity around the plaques was observed in the HE‐A, HE‐C, and HE‐S groups. Furthermore, a scatter plot analysis was conducted to assess the correlation between the clustered Iba‐1 fluorescence intensity and the area of each plaque (Figure 9C). Linear regression analysis indicated significant differences in the slopes of the regression equations for the HE‐A, HE‐C, and HE‐S groups compared with the vehicle group. The linear regression equations are as follows: Vehicle group: y = 633.8x + 276,179, *R* ^2^ = 0.60; HE‐A group: y = 363.6x + 60,219, *R* ^2^ = 0.42 (*p* < 0.0001 for slope difference); HE‐C group: y = 302.0x + 79,494, *R* ^2^ = 0.24 (*p* < 0.0001 for slope difference); and HE‐S group: y = 191.5x + 105,632, *R* ^2^ = 0.10 (*p* < 0.0031 for slope difference). These results demonstrate that the erinacines, specifically HE‐A, HE‐C, and HE‐S, diminish microglial activation surrounding plaques in a size‐dependent manner.

### 3.11 Erinacines Promote Neurogenesis and Secondary Branching of Immature Neural Dendrites in the Hippocampus of HFSTZ‐APP/PS1 Mice

**FIGURE 10:**
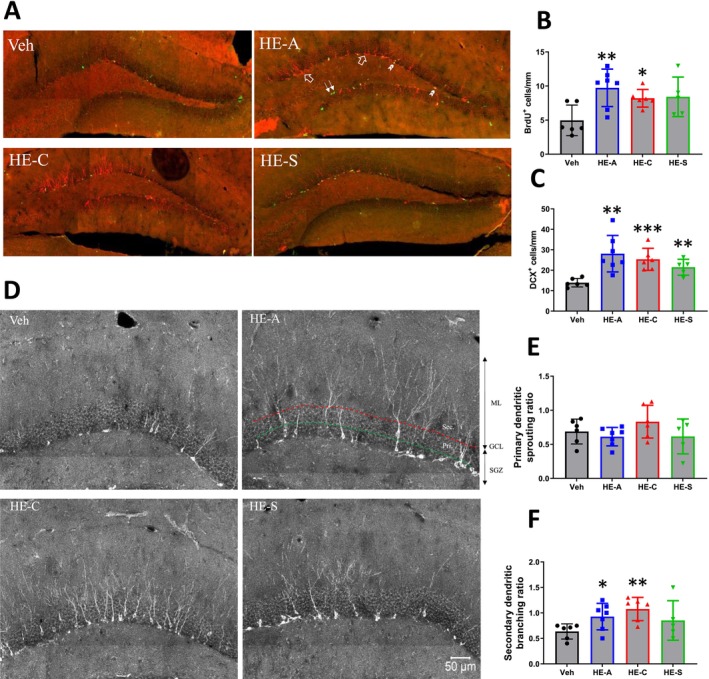
Erinacines promote neurogenesis and secondary branching of immature neural dendrites in the hippocampus of HFSTZ‐APP/PS1 mice. HFSTZ‐APP/PS1 mice were fed with vehicle (Veh), HE‐A (30 mg/kg), HE‐C (30 mg/kg), or HE‐S (30 mg/kg) (*n* = 6 for each group) and were injected with BrdU (50 mg/kg) for seven consecutive days before sacrifice, and brain sections were taken for tissue immunofluorescence staining. BrdU antibody was used to detect Type 2 neural progenitor (green), and Doublecortin (DCX) antibody was used to detect newborn neurons (red). Representative immunofluorescence staining of newborn neurons in the SGZ in the hippocampus (A). The arrows indicate BrdU^+^ Type2 neural progenitor, the open arrows indicate DCX^+^ new neurons, and the double arrows indicate BrdU^+^ DCX^+^ newborn neurons that have just completed differentiation. (B, C) Quantification of BrdU^+^ cells/mm (B) and DCX^+^ cells/mm (C) in the SGZ. The dendritic complexity was analyzed by laminar quantification (D). (E, F) Primary (pri.) and secondary (sec.) dendrites of the DCX^+^ cells are counted along the middle of GCL (green dashed line) and the outer edge of GCL (red dashed line), respectively. The sprouting ratio of the primary dendrites and the branching ratio of the DCX^+^ cells were shown. The results are presented as the mean ± SEM. Significant differences between the Veh group and the other groups are indicated by *p < 0.05, **p < 0.01, and ***p < 0.001. ML, molecular layer; GCL, granular cell layer; SGZ, subgranular zone.

We hypothesized that the reduction in amyloid plaques and microglial activation resulting from erinacine treatment might lead to improved hippocampal neurogenesis. To test this theory, we performed immunohistochemical staining with anti‐DCX and anti‐BrdU antibodies to evaluate the emergence of new granule neurons and the proliferation of type 2 progenitors within the SGZ. We observed an increase in the number of DCX‐positive new granule neurons in the SGZ of HFSTZ‐APP/PS1 mice treated with HE‐A, HE‐C, or HE‐S; similarly, the number of BrdU‐positive proliferating type 2 progenitors was elevated in the HE‐A and HE‐C groups (Figure 10A–C). Given the importance of dendritic growth for neuronal integration during neurogenesis, we further assessed the dendritic complexity of DCX‐positive cells using the laminar quantification method [40]. Our results indicated that HE‐A and HE‐C treatments significantly augmented the complexity of secondary dendritic branching (Figure 10D–F).

## 4 Discussion

Understanding microglial morphology and maturation is essential in AD research, as these cells are key to maintaining brain homeostasis. Microglia continuously monitor the brain environment, clearing debris and regulating synaptic function. A ramified microglial state is particularly important because it allows microglia to effectively detect and respond to subtle changes in the CNS, minimizing unnecessary inflammation and supporting neuronal health. Therefore, investigating how microglia alter their morphology and mature in response to potential treatments is critical for developing effective therapies for AD.

Erinacines, the bioactive compounds of Hericium erinaceus mushroom, have been reported to have a neuroprotective effect in APP/PS1 mice [27, 28]; however, their potential to modulate microglial morphology, elucidate underlying mechanisms of action, and address metabolic dysfunction in AD has not been thoroughly investigated. Our study is the first to demonstrate that erinacines can directly modulate microglia morphology, promoting a more ramified (surveillant) shape prior to activation by LPS. This highlights a previously unrecognized role of erinacines in maintaining microglial homeostasis, potentially contributing to their neuroprotective effects.

We developed a rat mixed glial culture model containing microglia and astrocytes that possesses physiological features that are missing from classic primary cultures. This model allowed us to reproduce the phenotypic changes in microglia when co‐cultured with astrocytes, enabling us to study the effects of erinacines on microglial morphology and phenotype. Earlier studies have indicated that the extent of branching in microglial cells correlates with their maturity, and an amoeboid shape suggests an immature phenotype [41]. In our primary mixed glial cultures, we observed a correlation between the ramified phenotype of microglia and the duration of culture in fresh medium. Microglial morphology assessment at DIV18 revealed an amoeboid‐like shape when the medium was refreshed at DIV17, allowing mixed glial cultures to persist in fresh medium for 1 day (DIFM1). Alternatively, medium refreshment at DIV14 allowed mixed glial cell cultures to transform in fresh medium for 4 days (DIFM4), resulting in a ramified morphology of microglia, suggesting an influence of culture duration on microglial phenotype. Activation of DIFM1‐microglia with LPS on DIV17 did not significantly alter morphology, yet heightened levels of pro‐inflammatory cytokines were detected in DIFM4‐microglia compared to DIFM1‐microglia, indicating a more mature state in DIFM4‐microglia. Interestingly, we found that HE‐A, HE‐Et, and HE‐S promoted the development of a ramified, mature microglial phenotype in DIFM1‐microglia. Moreover, HE‐A treatment of DIFM1‐microglia increased the expression of the M2 microglia marker CD206 and decreased the expression of the M1 microglia marker CD86, suggesting its potential to shift microglia toward a more neuroprotective state.

To reveal potential signaling pathways governing microglial phenotypic changes, we employed bioinformatic tools including CMap, LINCS, and L1000 [32, 33]. In this study, DIFM4, DIFM1, and DIFM1 treated with HE‐A, HE‐C, or HE‐S were subjected to L1000 gene expression profiling analysis. We first identified the underlying mechanisms that could reverse the DIFM1 gene profile back to DIFM4. The CMap results suggest that HDAC inhibition and bromodomain inhibition may be candidate pathways for the conversion of the DIFM1‐microglia phenotype to the DIFM4‐microglia phenotype. Moreover, from the gene expression profile, we noticed a twofold increase in the myelin basic protein (Mbp) in DIFM4‐microglia, which has been reported to be highly expressed in ramified microglia. Additionally, we observed a twofold increase of C‐X3‐C Motif Chemokine Receptor 1 (CX3CR1) in DIFM4‐microglia (Table S2). CX3CR1 is mostly expressed on microglia and is involved in maintaining the microglia in a quiescent state by binding to its ligand, fractalkine (CX3CL1), which is expressed in neurons [42]. Deleting of CX3CR1 on microglia decreases microglial ramification and maturation in young mice [43]. Additionally, a study showed that the absence of CX3CR1 impairs the microglial internalization of tau, contributing to the progression of AD [44].

To determine whether the effects of erinacines work through similar pathways, the gene expression of erinacine‐treated DIFM1 was compared to that of DIFM1. HDAC inhibition was identified as a key pathway, supported by our finding that erinacines closely resemble HDAC inhibitors in their mechanism of action. This is significant because HDAC inhibitors have been shown to promote microglial ramification [45], suggesting that erinacines might regulate microglial morphology through similar pathways. Treatment of DIFM1‐microglia with the HDAC inhibitors 11b and 11 h positively modulated microglia morphology, leading to a more ramified state. Interestingly, HDAC inhibitors could induce microglia ramification and suppress DIFM1‐microglia activation after adding LPS, suggesting that HDAC activity is directly involved in maintaining a less inflammatory, more ramified microglial phenotype. This result supports our hypothesis that erinacines might target HDAC activity for microglia ramifications. However, their increase in NO levels in DIFM1 microglia after LPS activation suggests that they might also be engaging other pathways that promote a more reactive microglial state in immature cells. This could be a part of their role in pushing microglia toward maturation, which might initially involve a transient increase in inflammatory mediators like NO. Thus, while HDAC inhibitors and erinacines both modulate microglial function, the unique effects of erinacines suggest that erinacines might influence microglia through a combination of HDAC inhibition and other, possibly maturation‐related, pathways. This highlights the complexity of microglial regulation and the potential of erinacines as a therapeutic agent with multifaceted mechanisms of action. Taken together, this result indicated that erinacines might target HDAC activity for microglia ramification; however, they might not specifically target HDACs for functional phenotypic changes. Future studies are required to investigate this possibility and to further elucidate the molecular pathways involved.

To further analyze the possible mechanism of action of erinacines, we recruited other bioinformatic systems such as CPDB and GSEA. Previous studies have indicated that the morphological change in microglia results from the rearrangement of cytoskeletal proteins, particularly actin filaments. The cells reconstruct the actin filaments for mobility and motility [46]. Moreover, Parakalan et al. showed that ramified microglia are enriched in genes that are mainly involved in cytoskeletal organization and cell differentiation [47]. Our CPDB results showed that HE‐A, HE‐C, and HE‐S shared upregulated genes that function in actin‐mediated cell contraction, cluster of actin‐based projections, and actin filament‐based movement. These changes likely contribute to the transformation of microglia from an amoeboid to a ramified state, further supporting the potential of erinacines to modulate microglial phenotype. Additionally, the regulation of lipid homeostasis and cholesterol metabolic processes by erinacines may have further implications for microglial function, as lipid metabolism is crucial for maintaining cellular membranes and supporting synaptic activity. Furthermore, our GSEA results indicated that erinacine treatments were associated with enhanced pathways supporting gliogenesis, glial cell differentiation, and microglial phagocytosis, further underscoring their potential role in modulating microglia to reach a more mature state.

Most AD patients are elderly and often suffer from metabolic disorders, making it essential to test potential treatments in models that more accurately reflect the human condition. Normal APP/PS1 mice do not fully capture the complexity of AD as it occurs in humans with metabolic stress. A high‐fat diet has been reported to increase Aβ deposition, activate microglia, and worsen memory in AD mice [48]. A high‐fat diet combined with streptozotocin (HFSTZ) has been shown to induce obesity‐associated oxidative stress, neuronal insulin resistance, glial cell activation, and neuroinflammation, which are important risk factors for neurodegeneration [22]. Although erinacines have been shown to be neuroprotective in normal APP/PS1 mice, metabolic stress can alter drug metabolism, distribution, and efficacy, leading to different therapeutic outcomes. In our study, we observed that erinacines, particularly HE‐A, exerted effects on metabolic regulation and clearance of Aβ. Erinacines not only reduce fasting blood glucose and insulin levels but also decrease amyloid‐beta accumulation and hyperactivated glial cells and substantially improved memory and behavioral deficits in HFSTZ‐APP/PS1 mice, as assessed by the nest‐building test. Additionally, our GSEA results revealed an increase in microglial phagocytosis following erinacine treatments, suggesting that erinacines might reduce amyloid plaques via enhancing microglia phagocytosis and reducing microglia‐mediated neuroinflammation. Our previous study also reported that HE‐My and HE‐Et may reduce Aβ through the augmented expression of insulin‐degrading enzyme (IDE), which is considered the possible mechanism by which erinacines reduce Aβ levels [27]. These findings underscore the potential of erinacines as a therapeutic option, especially for AD patients with metabolic disorders.

The role of microglia in AD extends beyond inflammation; they are also involved in neurogenesis and synaptic maintenance. In AD, the chronic inflammatory response driven by microglia contributes to impaired neurogenesis. Our study found that erinacines reduced plaque‐associated microglial and astrocytic activation, which in turn may have mitigated the inflammatory environment in the brain, allowing for enhanced neurogenesis. This was evidenced by increased proliferation of neural progenitors and improved dendritic branching in the hippocampus of HFSTZ‐APP/PS1 mice following erinacines treatments.

## 5 Conclusion

Our study highlights the therapeutic potential of *Hericium erinaceus* components, particularly erinacines, in promoting microglial ramification in vitro and mitigating Alzheimer's disease under conditions of metabolic stress in vivo. The ability of erinacines to shift microglia toward a more neuroprotective phenotype, reduce amyloid‐beta accumulation, and improve metabolic parameters underscores their promise as a multifaceted therapeutic approach. This study not only provides insight into the mechanisms by which erinacines modulate microglial morphology and function but also underscores their potential in targeting the complex interplay between metabolic stress and neurodegeneration in AD. These findings suggest that erinacines are the promising candidates for the prevention and treatment of AD, especially for patients with comorbid metabolic disorders, thereby addressing an unmet clinical need in the treatment of neurodegenerative diseases.

## Author Contributions

Van Thanh Bui and Kuan‐Wei Wu performed the experiments and data analysis, and participated in the drafted manuscript. Anh Thuc Nguyen performed the gene set enrichment analysis. Chin‐Chu Chen, Li‐Ya Lee, and Wan‐Ping Chen participated in the fermentation of *H. erinaceus* mycelia and performed the isolation and component identification of *H. erinaceus* mycelia. Wei‐Jan Huang provided compounds 11b and 11h. Young‐Ji Shiao and Chi‐Ying Huang participated in the study design and coordination and helped to draft the manuscript. All the authors read and approved the final manuscript.

## Ethics Statement

The authors have nothing to report.

## Consent

All authors approve publication.

## Conflicts of Interest

The authors declare no conflicts of interest.

## Supporting information


Figure S1.
Figure S2.
Figure S3.

Table S1.
Table S2.
Table S3.
Table S4.

## Data Availability

The datasets used and/or analyzed during the current study are available from the corresponding author on reasonable request.
