# Supplementary material for: Exploring the Synergistic Effects of Erinacines on Microglial Regulation and Alzheimer's Pathology Under Metabolic Stress
Source: CNS Neurosci Ther. 2024 Dec 17;30(12):e70137. doi: 10.1111/cns.70137 (PMC11652784; doi:10.1111/cns.70137)
Supplement: Supplementary file 1 — Figure S1. Figure S2. Figure S3. [file CNS-30-e70137-s001.docx]

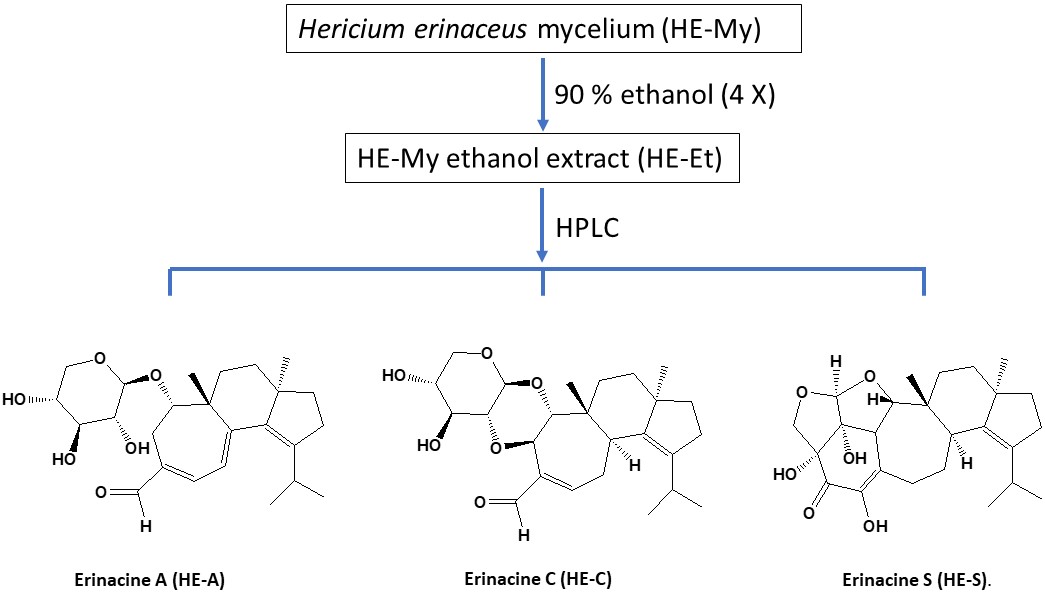


**Supplementary Figure 1**. The preparation and structure of erinacine A (HE-A), erinacine C (HE-C), and erinacine S (HE-S).


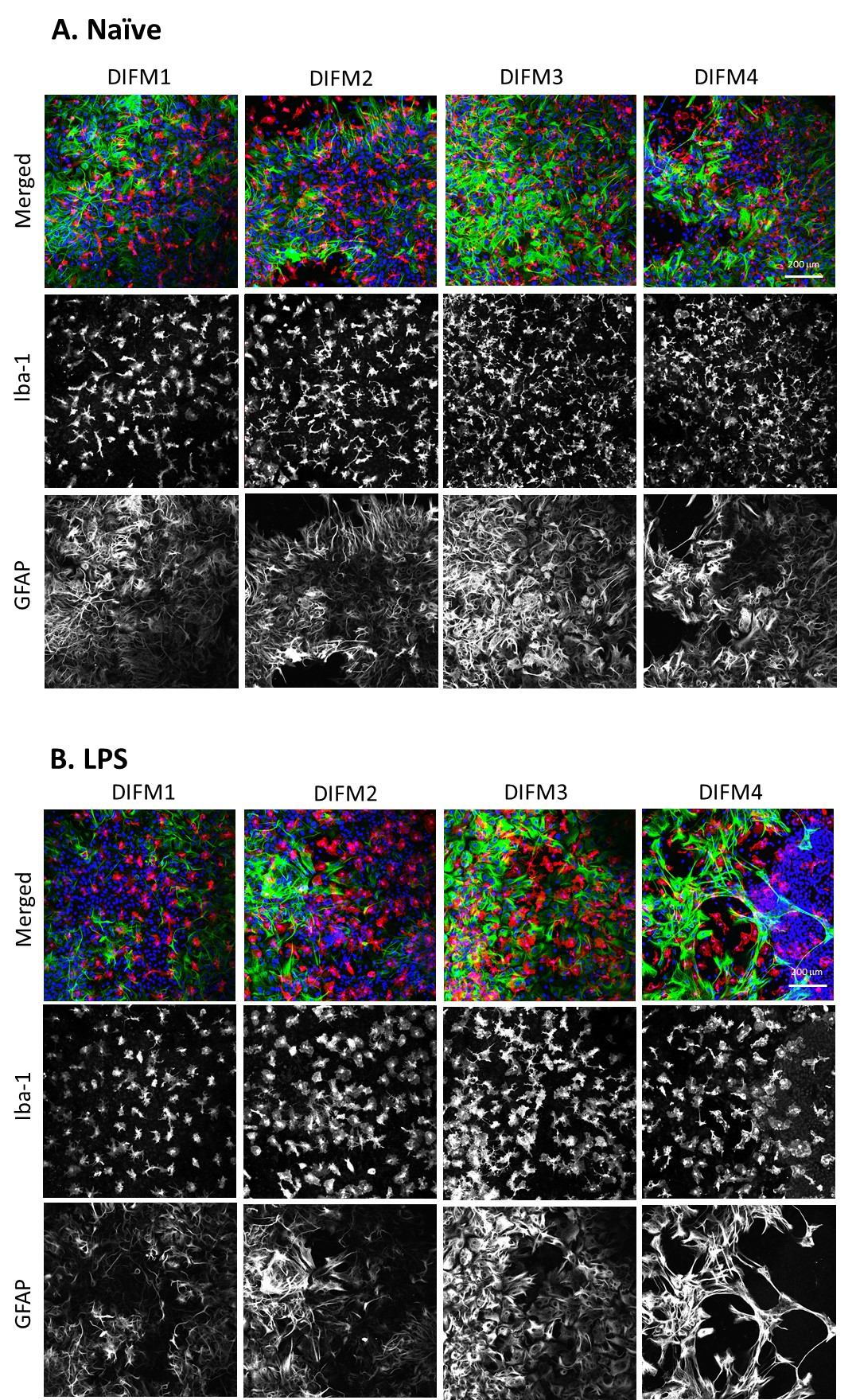


**Supplementary Figure 2. Using mixed glial cultures to study microglial maturation/ramification.** Morphological Changes of Microglia Dependent on Days in Fresh Medium (DIFM). After refreshing and treatment, cells were fixed and subjected to immunostaining of microglia by anti-Iba1 antibody (red, and black in middle panel), anti-GFAP antibody (green). Nucleus were stained with Hoechst33258 (blue, and black in lower panel)). The representative image of the naïve cells (A) and the cell treated with LPS (B) was shown.

**
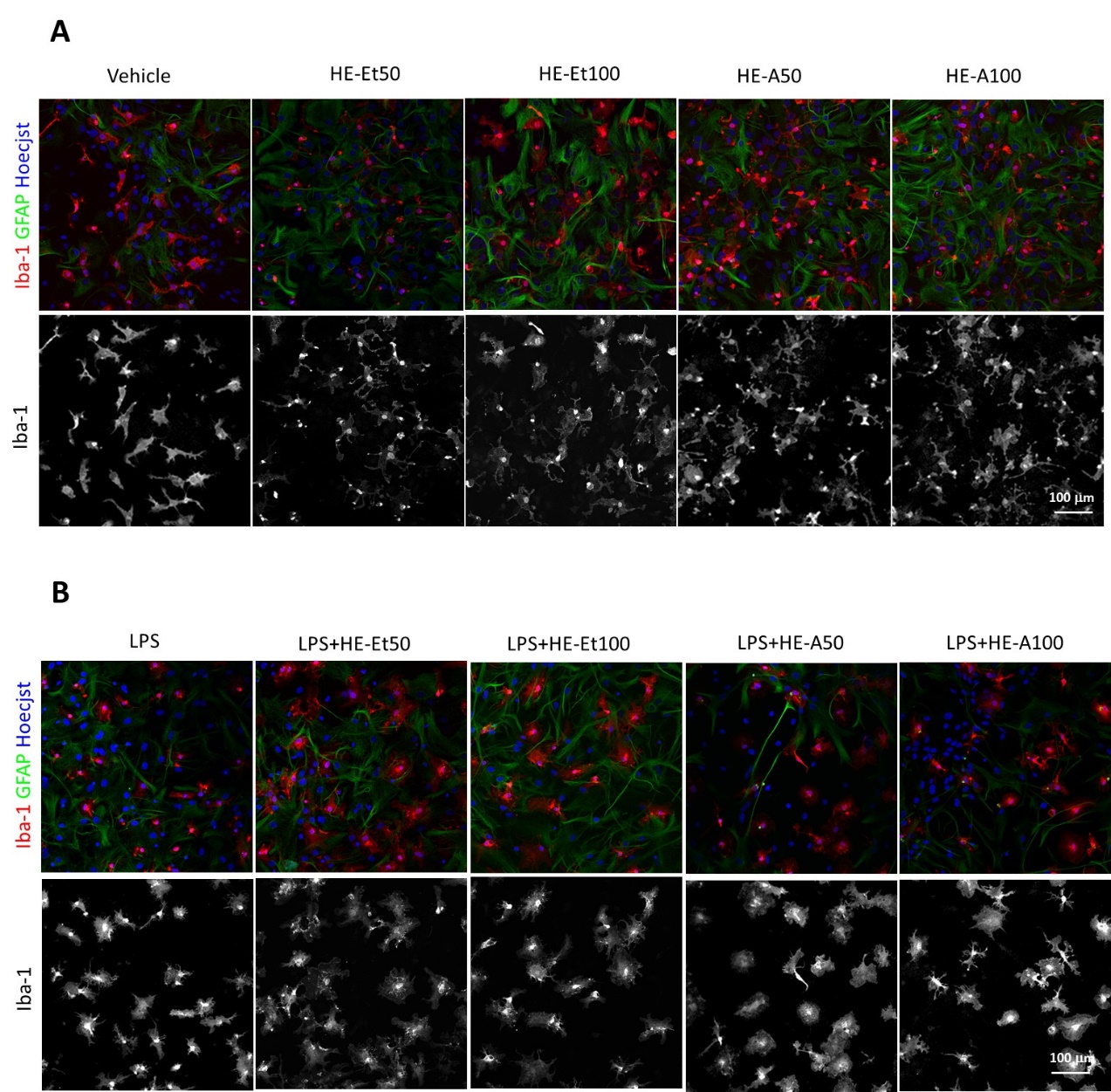
**

**Supplementary Figure 3. The effects of HE-Et and HE-A on microglial maturation/ramification.** After treatment, cells were fixed and subjected to immunostaining of microglia by anti-Iba1 antibody (red, and black in lower panel), anti-GFAP antibody (green). Nucleus were stained with Hoechst33258 (blue). The representative image of the naïve cells (A) and the LPS-treated cells (B) was shown.

**
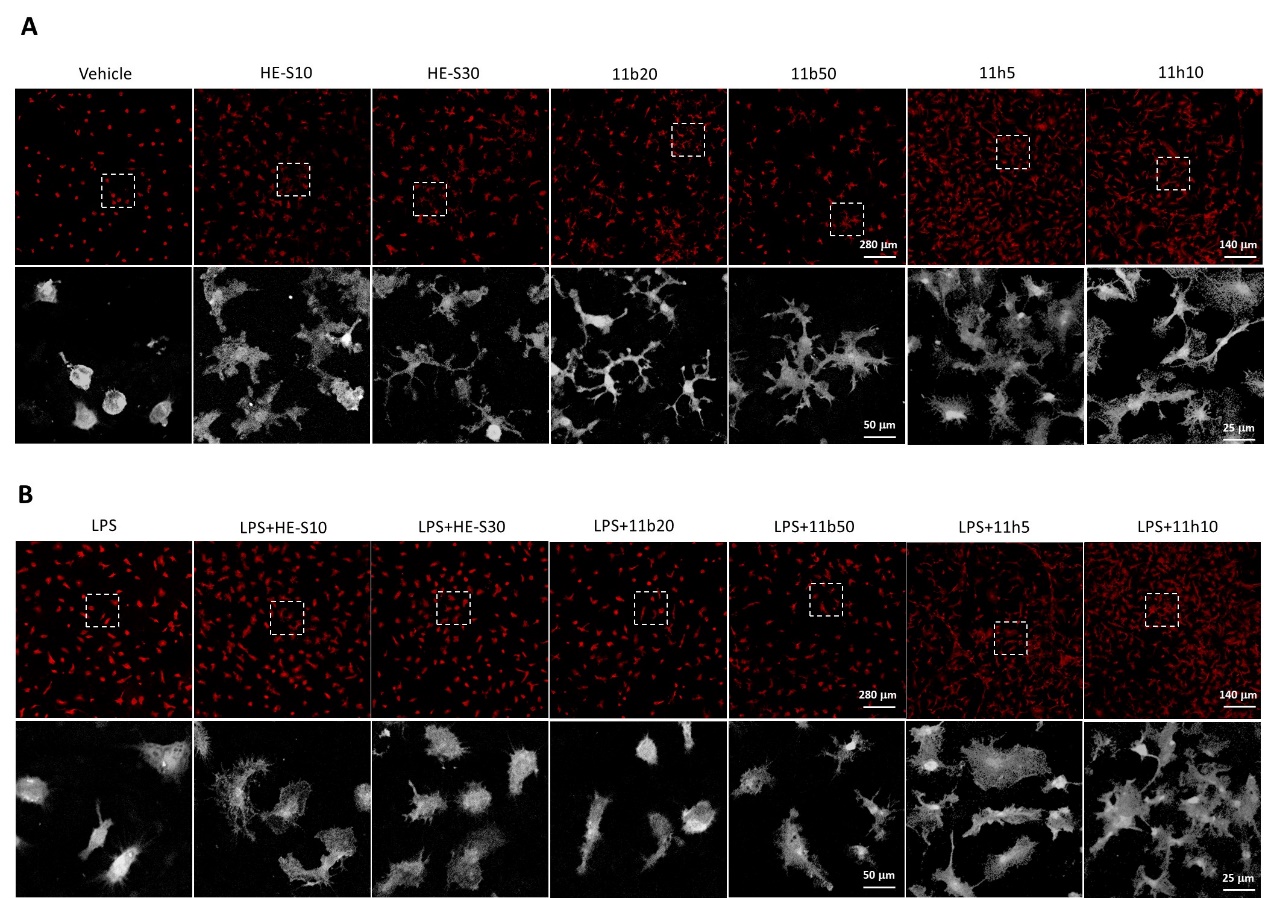
**

**Supplementary Figure 4. The effects of HE-S, 11b, and 11h on microglial maturation/ramification.** After treatment, cells were fixed and subjected to immunostaining of microglia by anti-Iba1 antibody. The representative image of the naïve cells (A) and the LPS-treated cells (B) was shown and magnified images of the box area.
