# Supplementary material for: Exploring the Synergistic Effects of Erinacines on Microglial Regulation and Alzheimer's Pathology Under Metabolic Stress
Source: CNS Neurosci Ther. 2024 Dec 17;30(12):e70137. doi: 10.1111/cns.70137 (PMC11652784; doi:10.1111/cns.70137)
Supplement: Supplementary file 2 — Table S1. Table S2. Table S3. Table S4. [file CNS-30-e70137-s002.docx]

**Supplementary Table 1. CMap result: Perturbagen compound (PC) of DIFM4 group.** The PC, description of the compound, perturbagen score, and common functions to other groups were indicated.

| **PC** | **Description** | **Score** | **Common to** |
| --- | --- | --- | --- |
| PJ-34 | PARP inhibitor | 98.92 |  |
| ochratoxin-a | PheRS inhibitor | 98.38 |  |
| U-0126 | MEK inhibitor | 98.36 |  |
| PIK-75 | DNA protein kinase inhibitor | 98.06 |  |
| chromomycin-a3 | DNA binding agent | 98.06 |  |
| ISOX | HDAC inhibitor | 98.06 | HE-S, -C |
| ZG-10 | JNK inhibitor | 97.96 | HE-S, -C |
| tivozanib | VEGFR inhibitor | 97.89 |  |
| AS-605240 | PI3K inhibitor | 97.82 |  |
| triptolide | RNA polymerase inhibitor | 97.82 |  |
| scriptaid | HDAC inhibitor | 97.78 | HE-S, -C |
| mitoxantrone | Topoisomerase inhibitor | 97.73 |  |
| pirarubicin | Topoisomerase inhibitor | 97.71 |  |
| ER-27319 | Mediator release inhibitor | 97.67 |  |
| pidorubicine | Topoisomerase inhibitor | 97.57 |  |
| belinostat | HDAC inhibitor | 97.21 | HE-S, -C |
| daunorubicin | RNA synthesis inhibitor | 96.95 |  |
| idarubicin | Topoisomerase inhibitor | 96.72 |  |
| trichostatin-a | HDAC inhibitor | 96.48 | HE-S, -C |
| TG-101348 | FLT3 inhibitor | 96.3 |  |
| proxyfan | Histamine receptor modulator | 96.23 |  |
| AS-703026 | MEK inhibitor | 96.19 |  |
| Bisindolylmaleimide-ix | CDK inhibitor | 96.00 |  |
| dasatinib | BCR-ABL kinase inhibitor | 95.95 |  |
| alvocidib | CDK inhibitor | 95.80 |  |
| givinostat | HDAC inhibitor | 95.79 |  |
| vorinostat | HDAC inhibitor | 95.60 | HE-S, -C |
| AZ-628 | RAF inhibitor | 95.52 |  |
| PP-2 | SRC inhibitor | 95.51 |  |
| XMD-892 | MAP kinase inhibitor | 95.45 | HE-A |
| TPCA-1 | IKK inhibitor | 95.45 |  |
| THM-I-94 | HDAC inhibitor | 95.14 | HE-S, -C |
| PF-562271 | Focal adhesion kinase inhibitor | 94.93 |  |
| apicidin | HDAC inhibitor | 94.57 | HE-C |
| geldanamycin | HSP inhibitor | 94.47 |  |
| NVP-AUY922 | HSP inhibitor | 94.42 |  |
| NCH-51 | HDAC inhibitor | 94.29 | HE-C |
| CGP-60474 | CDK inhibitor | 93.83 |  |
| dactinomycin | RNA polymerase inhibitor | 93.69 |  |
| dacinostat | HDAC inhibitor | 93.66 |  |
| XMD-885 | LRRK inhibitor | 93.59 |  |
| vemurafenib | RAF inhibitor | 93.56 | HE-S |
| azithromycin | Bacterial 50S ribosomal subunit inhibitor | 93.36 |  |
| PP-30 | RAF inhibitor | 93.32 |  |
| BMS-536924 | IGF-1 inhibitor | 93.08 |  |
| VER-155008 | HSP inhibitor | 92.77 |  |
| HC-toxin | HDAC inhibitor | 92.57 |  |
| PI-828 | PI3K inhibitor | 92.57 |  |
| AT-7519 | CDK inhibitor | 92.55 |  |
| BI-2536 | PLK inhibitor | 92.42 |  |
| doxorubicin | Topoisomerase inhibitor | 92.17 |  |
| promazine | Dopamine receptor antagonist | 91.96 |  |
| XMD-1150 | Leucine rich repeat kinase inhibitor | 91.81 |  |
| maprotiline | Norepinephrine reuptake inhibitor | 91.36 |  |
| GSK-1059615 | PI3K inhibitor | 91.30 |  |
| pyroxamide | HDAC inhibitor | 91.28 | HE-S |
| ellipticine | Topoisomerase inhibitor | 91.00 |  |
| HG-5-113-01 | Protein kinase inhibitor | 90.75 |  |
| BIBX-1382 | EGFR inhibitor | 90.66 |  |
| tubastatin-a | HDAC inhibitor | 90.59 |  |

**BCR-ABL,** breakpoint cluster region-Abelson; **CDK,** cyclin-dependent kinase; **EGFR,** epidermal growth factor receptor; **FLT3,** FMS-like tyrosine kinase 3; **HDAC,** histone deacetylase; **HSP,** heat shock protein; **IGF-1,** Insulin-Like Growth Factor –1; **IKK,** IκB kinase; **JNK,** c-jun N-terminal kinase; **LRRK,** leucine-rich repeat kinase; **MAP,** mitogen-activated protein**; MEK,** MAP/ERK kinase; **PARP,** Poly (ADP-ribose) polymerase; **PheRS,** Phenylalanyl tRNA synthetase; **PI3K,** phosphoinositide 3-kinase; **PLK,** Polo-like kinase; **SRC,** sarcoma; **VEGFR,** vascular endothelial growth factor.

**Supplementary Table 2. Up-regulated genes in DIFM4 and the genes common to the genes in DIFM1 treated with HE-A, HE-S and HE-C.**

| **Gene** | **Description** | **Fold** | **Common to** |
| --- | --- | --- | --- |
| Tgfbi | RGD-containing protein that binds to collagens. | 3.9 |  |
| Pdgfrb | Cellular migration and proliferation. | 3.22 |  |
| Coro1a | helping phagocytosis. | 2.94 |  |
| Suco | Collagen synthesis during postnatal maturation. | 2.82 |  |
| Cadm2 | Cell adhesion. | 2.81 |  |
| Thbs1 | Platelet aggregation, angiogenesis, and tumorigenesis. | 2.75 | HE-S |
| Rlf | DNA hypomethylation, epigenetic gene silencing. | 2.74 | HE-A, -C |
| Osr1 | A zinc-finger transcription factor. | 2.73 |  |
| Tnks2 | Inhibits TERF1 bind to telomeric DNA | 2.7 | HE-A |
| Cdh13 | Dynamic cytoskeleton reorganization. | 2.69 |  |
| Rbp1 | Transport of retinol from the liver to peripheral tissue. | 2.67 | HE-A, -S, -C |
| Nudt21 | mRNA processing binding Reactome. | 2.63 | HE-C |
| Foxc1 | The regulation of embryonic and ocular development. | 2.61 |  |
| Cdkn1b | Bind Cyclin and Cdk. | 2.6 |  |
| Top2b | Recombination or supporting transcription in neurons | 2.6 |  |
| Tpm2 | Critical to normal cardiac function | 2.59 | HE-A, -S, -C |
| Slmap | Defining the individual muscle fiber. | 2.59 |  |
| Baz1b | Chromatin-dependent regulation of transcription. | 2.5 |  |
| Psat1 | Cell proliferation, survival, migration and epigenetics. | 2.48 |  |
| Atrx | Maintaining silencing at telomeres. | 2.45 |  |
| Sypl2 | Interacts with synaptobrevin. | 2.44 |  |
| Baz2b | Chromatin-dependent regulation of transcription. | 2.43 |  |
| Pld2 | Cleavage of the phosphodiester bond in phospholipids. | 2.42 |  |
| Sema3c | Axonal growth cone guidance molecules. | 2.4 |  |
| Pnmal2 | Trigger an autoimmune response. | 2.4 |  |
| Sri | Response to cellular stress in neurodegeneration. | 2.38 | HE-A |
| Rbms3 | It significantly associated in breast cancer. | 2.38 |  |
| Smc2 | Participate in chromosome organization and dynamics. | 2.37 |  |
| Nrp1 | Angiogenesis, axon guidance, and migration. | 2.36 |  |
| Dab1 | The migration and differentiation of neurons. | 2.35 | HE-A, -C |
| Tnrc18 | Assist in the silencing of genes. | 2.35 |  |
| Pcmtd1 | A putative E3 ubiquitin ligase substrate adaptor protein. | 2.34 |  |
| Ddx3y | An immunogenic protein. | 2.3 |  |
| Slc44a2 | Movement of molecules across a biological membrane. | 2.3 |  |
| Lats2 | Mediator of the cell stress response pathways. | 2.3 |  |
| Maf | A transcription factor of the b-Zip family. | 2.3 |  |
| Ubqln2 | Ubiquitin-like protein. | 2.27 |  |
| Pmm1 | GDP-mannose synthesis. | 2.25 | HE-A, -C |
| Sf3a3 | Subunit 3 of the splicing factor 3a protein complex. | 2.25 | HE-A, -S, -C |
| Kif5b | Intracellular movement and cytoplasmic transport. | 2.24 |  |
| Rapgef6 | Protein localization to plasma membrane. | 2.24 |  |
| Dzip1 | Required for ciliogenesis in cultured mammalian cells. | 2.22 | HE-C, -S |
| Stox2 | Pre-eclampsia with fetal growth restriction. | 2.22 |  |
| Nog | Promoting somite patterning in the developing embryo. | 2.21 |  |
| Mbp | Myelination of nerves in the nervous system. | 2.21 |  |
| Ralgapa1 | Regulation of transcription, DNA-templated. | 2.21 |  |
| Uty | The immunity-driven susceptibility to complex disease. | 2.2 |  |
| Hacd4 | Oxidoreductase. | 2.2 |  |
| Nrg2 | Induces the growth and differentiation of cells. | 2.19 | HE-A, -C |
| Baz2b | Recognize and bind to acetylated Lys residues. | 2.19 |  |
| Aldh1a2 | Participate in detoxification of aldehydes. | 2.18 |  |
| Sh3kbp1 | Enhances TNF-mediated apoptotic cell death. | 2.18 |  |
| Sostdc1 | Cellular proliferation, differentiation, and apotosis. | 2.18 |  |
| Epha4 | Mediating developmental events in the nervous system. | 2.17 |  |
| Capn12 | Participants in cell mobility and cell cycle progression. | 2.17 | HE-A, -S, -C |
| Arid5b | Regulates the transcription in adipogenesis. | 2.16 |  |
| Prkar1b | A regulatory subunit of PKA. | 2.15 | HE-S |
| Cp | Carries of the copper in plasma. | 2.15 |  |
| Coro1a | Helping phagocytosis. | 2.14 |  |
| Vegfc | Promote lymphangiogenesis via VEGFR-3. | 2.14 |  |
| Chd2 | Catalyzes the assembly of chromatin into periodic arrays. | 2.13 |  |
| Hdlbp | The regulation of gene expression. | 2.12 | HE-S |
| Dbp | Binds to an upstream promoter in the insulin gene. | 2.12 |  |
| Cald1 | A calmodulin- and actin-binding protein that plays an essential role in the regulation of smooth muscle and nonmuscle contraction. | 2.12 |  |
| Znrd1as | Subunit of RNA polymerase I. | 2.12 |  |
| Slc13a3 | Transport Krebs cycle intermediates. | 2.12 | HE-A |
| Ncapd3 | Chromosome assembly and segregation. | 2.12 | HE-A, -S, -C |
| Atp1a2 | Maintaining the electrochemical gradients of Na^+^/K^+^. | 2.12 | HE-A |
| Rgcc | Interphase and mitosis. | 2.11 |  |
| Rai2 | Wnt signaling in multiple and complex ways. | 2.11 |  |
| Anks1b | It interacts with amyloid beta protein precursor. | 2.1 |  |
| Il16 | A chemoattractant, a modulator of T cell activation. | 2.09 |  |
| Lims1 | Involved in focal adhesion plaques. | 2.09 |  |
| Lima1 | Embryonic development and cell lineage determination. | 2.07 | HE-C |
| Twf2 | Actin-monomer-binding protein. | 2.07 |  |
| Jak1 | Essential for signaling for certain cytokines. | 2.07 |  |
| Cx3cr1 | Migration, adhesion and retention of leukocytes. | 2.07 | Quiescent gene |
| Itga4 | Belongs to the integrin alpha chain family of proteins. | 2.07 |  |
| C1ql3 | Regulates insulin secretion from pancreatic β-cells. | 2.05 |  |
| Sh3rf3 | Predicted to enable ubiquitin protein ligase activity. | 2.05 |  |
| Pla2g15 | Regulate the multifunctional lysophospholipids. | 2.04 | HE-A, -S |
| Ptprd | Regulate cell growth, differentiation, and mitotic cycle. | 2.04 |  |
| Prpf4b | Pre-mRNA splicing and in signal transduction. | 2.04 |  |
| Fzd2 | Involved in transmembrane signal transmission. | 2.03 |  |
| Ogfrl1 | Important in embryonic development and wound repair. | 2.03 | HE-A, -C |
| Tbl1x | Involved in cytoskeletal assembly. | 2.03 |  |
| Lrrtm1 | Development of specific forebrain structures. | 2.03 |  |
| Dntt | Adding nucleotides to the 3' terminus of a DNA. | 2.03 |  |
| Atr | Sensing DNA damage leading to cell cycle arrest. | 2.03 |  |
| Spg20 | Endosomal trafficking and microtubule dynamics. | 2.02 |  |
| Rreb1 | Cell proliferation/transcription and DNA damage repair. | 2.02 |  |
| BC024139 | Regulation of focal adhesion assembly. | 2.02 | HE-A, -S, -C |
| Agl | Involves in glycogen degradation. | 2.01 |  |
| Nupr1 | Various events in response to various cellular stressors. | 2 |  |
| Mef2c | Transcription factor in the Mef2 family. | 2 |  |
| Shc1 | Control T cell development and activation. | 2 |  |

**RGD,** Arg-Gly-Asp; **TERF1,** telomeric repeat binding factor 1; **cdk,** cyclin-dependent kinase; **Mef2,** myocyte enhancer factor 2; **TNF,** tumor necrotic factor; **PKA,** protein kinase A; **VEGFR,** vascular endothelial growth factor.

**Supplementary Table 3. down-regulated genes in DIFM4 and the genes common to the genes in DIFM1 treated with HE-A, HE-S and HE-C.**

| **Gene** | **Description** | **Fold** | **Common to** |
| --- | --- | --- | --- |
| Ccl3 | Plays a role in inflammatory responses. | -4.79 |  |
| Nfkbiz | Nuclear inhibitor of NF-κB (IκB) protein. | -4.49 |  |
| Zbtb16 | Involved in cell cycle progression. | -3.8 | HE-C |
| Ntng1 | Axon guidance during nervous system development. | -3.71 |  |
| Fosl1 | Induced by Ras-ERK or PI3K-AKT pathway. | -3.19 |  |
| Ptx3 | Involved in complement activation and amplification. | -3.07 |  |
| Egr3 | Induced by mitogenic stimulation. | -2.78 |  |
| Spcs1 | Peptidase activity and ribosome binding activity. | -2.69 | HE-A, -S, -C |
| Igh-V7183 | The large polypeptide subunit of an antibody. | -2.65 | HE-A, -S, -C |
| Cxcl1 | Chemokine. | -2.57 |  |
| Nfkbia | Nuclear inhibitor of NF-κB (IκB) protein. | -2.43 |  |
| Ddit4l | Inhibits activation of the mTORC-1, leading to cell death. | -2.41 |  |
| Crlf1 | Promotes survival of neuronal cells. | -2.37 |  |
| Riok1 | Essential in the maturation of 40S subunits. | -2.36 | HE-A, -S, -C |
| Calr3 | Binds to misfolded proteins and prevents exportation. | -2.34 | HE-A, -S, -C |
| Irf1 | A transcription factor for expression of the IFNβ. | -2.26 | HE-A |
| Gna14 | Plays a role in activation of PLC-β. | -2.26 | HE-A, -S, -C |
| Trip13 | Hormone-dependent transcription factors. | -2.22 | HE-A, -S, -C |
| Bcl2a1a/b/d | Negative regulation of apoptotic process. | -2.21 |  |
| Btg1 | Coactivator of cell differentiation. | -2.17 |  |
| Dsg2 | Involved in cell-cell junctions between cells. | -2.15 |  |
| Ndn | Suppresses growth in postmitotic neurons. | -2.14 | HE-A, -C |
| Plpp6 | Involved in phospholipid dephosphorylation. | -2.12 | HE-A, -S, -C |
| Boc | Axon guidance and smoothened signaling pathway. | -2.11 | HE-A, -S, -C |
| Ube2z | Protein ubiquitination for protein degradation machinery. | -2.11 | HE-A, -C |
| Mapre1 | A microtubule plus end tracking protein. | -2.07 | HE-A |
| Gm38396 | Sphingomyelin phosphodiesterase. | -2.06 | HE-C |
| Cebpb | Interacts with the CCAAT box in several gene promoters. | -2.05 |  |
| Cyp4a31 | Fatty acid dehydrogenase and monooxygenase activity. | -2.04 | HE-A, -S, -C |
| Sh2b1 | Function in cytokine and growth factor receptor signaling. | -2.03 |  |
| Dcaf12l1 | Function in cell cycle, apoptosis, and gene regulation. | -2.03 | HE-A, -S, -C |
| Tfap2a | Enhances transcription by binding to a GC-rich sequence. | -2.02 | HE-S |

**ERK,** extracellular signal-regulated kinases; **IFNβ,** interferon β; **IκB,** nuclear factor of kappa light polypeptide gene enhancer in B-cells inhibitor; **mTORC-1,** mechanistic target of rapamycin complex 1; **NF-κB,** nuclear factor kappa-light-chain-enhancer of activated B cells; **PI3K,** phosphoinositide 3-kinase; **PLC-β,** phospholipase C-β.

**Supplementary Table 4. The biological processes and molecular functions involved in the activities of erinacines and DIFM4 transformation.** The up-regulated genes are indicated in blue and the down-regulated genes are indicated in red.

| GO:0051220 | Cytoplasmic sequestering of protein | The selective interaction of a protein with specific molecules in the cytoplasm, thereby inhibiting its transport into other areas of the cell. |
| --- | --- | --- |
| GO:0045185 | **Maintenance of protein location** | Any process in which a protein is maintained in a location and prevented from moving elsewhere. These include sequestration, stabilization to prevent transport elsewhere and the active retrieval of proteins that do move away. |
| GO:0005903 | Brush border | The dense covering of microvilli on the apical surface of an epithelial cell in tissues such as the intestine, kidney, and choroid plexus; the microvilli aid absorption by increasing the surface area of the cell. |
| GO:0070252 | **Actin-mediated cell contraction** | The actin filament-based process in which cytoplasmic actin filaments slide past one another resulting in contraction of all or part of the cell body." |
| GO:0030048 | Actin filament-based movement | Movement of organelles or other particles along actin filaments, or sliding of actin filaments past each other, mediated by motor proteins. |
| GO:0008203 | Cholesterol metabolic process | The chemical reactions and pathways involving cholesterol, cholest-5-en-3 beta-ol, the principal sterol of vertebrates and the precursor of many steroids, including bile acids and steroid hormones. It is a component of the plasma membrane lipid bilayer and of plasma lipoproteins and can be found in all animal tissues. |
| GO:0055088 | Lipid homeostasis | Any process involved in the maintenance of an internal steady state of lipid within an organism or cell. |
| GO:0098862 | Cluster of actin-based cell projections | A cell part consisting of multiple, closely packed actin-based cell projections. |
| GO:0016125 | Sterol metabolic process | The chemical reactions and pathways involving sterols, steroids with one or more hydroxyl groups and a hydrocarbon side-chain in the molecule. |
| GO:1902494 | Catalytic complex | A protein complex which is capable of catalytic activity |
| GO:0016787 | Hydrolase activity | Catalysis of the hydrolysis of various bonds, e.g., C-O, C-N, C-C, phosphoric anhydride bonds, etc. |
| GO:0035639 | Purine nucleoside triphosphate binding | Binding to ATP, GTP, XTP, and ITP, etc.. |
| GO:0032555 | Purine ribonucleotide binding | Binding to ATP, ADP, AMP, GTP, GDP, and GMP, etc. |
| GO:0017076 | Purine nucleotide binding | Binding to ATP, ADP, AMP, GTP, GDP, GMP, dATP, and dGTP, etc. |
| GO:0032553 | Ribonucleotide binding | Binding to AMP, GMP, UMP, and CMP, etc. |
| GO:005515 | Protein binding | Binding to a protein. |
| GO:0015631 | Tubulin binding | Binding to monomeric or multimeric forms of tubulin, including microtubules. |
| GO:0001228 | DNA-binding transcription activator activity, RNA polymerase II-specific | A DNA-binding transcription factor activity that activates or increases transcription of specific gene sets transcribed by RNA polymerase II. |
